# Health Resource Utilization and Outcomes Among Patients Who Receive Extracorporeal Membrane Oxygenation

**DOI:** 10.1016/j.cjco.2025.03.019

**Published:** 2025-03-31

**Authors:** Vicki Papanikolaou, Ethan Goh, Alayna Carrandi, Anaïs Charles-Nelson, Kiran Kottakkal, Lorena Romero, Carol L. Hodgson, Alisa M. Higgins

**Affiliations:** a Australian and New Zealand Intensive Care Research Centre, School of Public Health and Preventive Medicine, Monash University, Melbourne, Victoria, Australia; b Alfred Health, Melbourne, Victoria, Australia

## Abstract

**Background:**

Extracorporeal membrane oxygenation (ECMO) is a high-cost, resource-intensive intervention for severe cardiac and respiratory failure. Survivors of ECMO have healthcare needs that extend beyond their acute hospitalization, placing significant demands on healthcare systems and society. As ECMO use increases globally, understanding the healthcare and economic burden of ECMO survivorship is needed to improve long-term outcomes of ECMO survivors and optimize resource allocation within healthcare systems.

**Methods:**

We systematically searched Ovid MEDLINE, Ovid Embase, Ovid Emcare, Scopus, and Web of Science from inception to October 1, 2024. We included studies published in English that reported post-discharge healthcare resource utilization and costs for adult survivors of ECMO.

**Results:**

From 1889 articles screened, 24 studies met our inclusion criteria. Most were retrospective cohort studies conducted in North America between 2010 and 2024. Sample sizes ranged from 12 to 23,876 patients, with in-hospital mortality rates between 28.5% and 67.4%. Thirty-day readmission rates ranged from 10.0% to 43.8%, with 90-day rates ranging from 21.1% to 53.0%. One study reported a mean 30-day readmission cost of $62,150 (in 2023 US dollars). Only 5 studies (20.8%) reported total healthcare costs up to 6 months and 1 year.

**Conclusions:**

ECMO survivorship is gaining increased attention in research and clinical practice, yet significant gaps remain in our understanding of long-term healthcare resource utilization and costs. Our review reveals very limited information, indicating an urgent need for more comprehensive and granular data to guide clinical and policy decisions and achieve healthcare system efficiency.

## Graphical abstract

**Figure:**
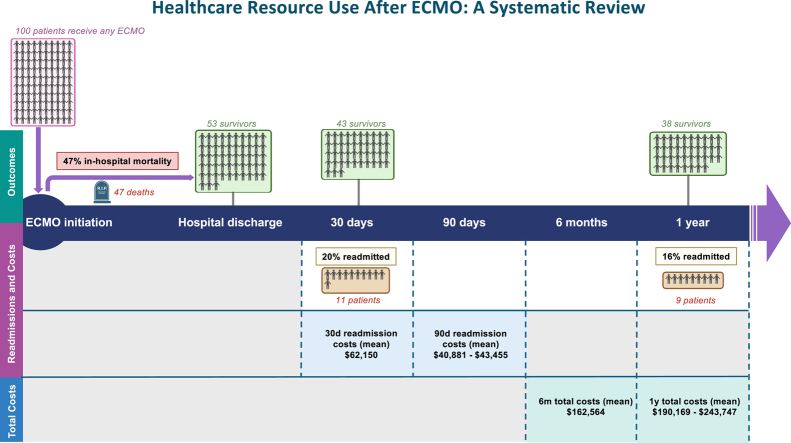
Hypothetical cohort of patients undergoing ECMO, showing survival at hospital discharge, 30 days, and 1 year, along with hospital readmissions during follow-up. Patients classified as ‘readmitted’ had at least one readmission during the follow-up period

## Résumé

**Contexte:**

L'oxygénation extracorporelle par membrane (ECMO) est une intervention coûteuse et gourmande en ressources pour traiter les insuffisances cardiaques et respiratoires graves. Les survivants de l'ECMO ont des besoins en matière de soins de santé qui vont au-delà de leur hospitalisation aiguë, ce qui impose des contraintes importantes aux systèmes de soins de santé et à la société. Alors que l'utilisation de l'ECMO augmente à l'échelle mondiale, il est nécessaire de comprendre le fardeau sanitaire et économique de la survie après ECMO pour améliorer les résultats à long terme des survivants de l'ECMO et ainsi optimiser l'allocation des ressources au sein des systèmes de soins de santé.

**Méthodologie:**

Nous avons effectué des recherches systématiques sur les bases de données Ovid Medline, Ovid Embase, Ovid Emcare, Scopus et Web of Science depuis le début jusqu'au 1^er^ octobre 2024. Nous avons inclus les études publiées en anglais qui faisaient état de l'utilisation des ressources de soins de santé après la sortie de l'hôpital et des coûts pour les adultes ayant survécu à une ECMO.

**Résultats:**

Sur les 1 889 articles examinés, 24 études répondaient à nos critères d'inclusion. La plupart étaient des études de cohortes rétrospectives menées en Amérique du Nord entre 2010 et 2024. La taille des échantillons variait de 12 à 23 876 patients, avec des taux de mortalité à l'hôpital compris entre 28,5 % et 67,4 %. Les taux de réadmission à 30 jours allaient de 10,0 % à 43,8 %, et les taux à 90 jours de 21,1 % à 53,0 %. Une étude révélait un coût moyen de réadmission à 30 jours de 62 150 $ ($ US de 2023). Seules cinq études (20,8 %) rapportaient les coûts totaux des soins de santé jusqu'à 6 mois et 1 an.

**Conclusion:**

La recherche et la pratique clinique s'intéressent de plus en plus à la survie des patients après ECMO, mais des lacunes importantes subsistent dans notre compréhension de l'utilisation des ressources et des coûts des soins de santé à long terme. Notre revue de littérature a révélé des informations très limitées, indiquant un besoin urgent de données plus complètes et plus granulaires pour guider les décisions cliniques et politiques et atteindre l'efficacité du système de soins de santé.

## Introduction

Extracorporeal membrane oxygenation (ECMO) is a high-cost, resource-intensive intervention used to support patients with severe cardiac and respiratory failure.^1^^,^^2^ Typically reserved for patients unresponsive to conventional treatments and at high risk of mortality, ECMO provides temporary circulatory or respiratory support to facilitate recovery or serve as a bridge to device implantation or organ transplantation.^3^^,^^4^ In addition to its lifesaving potential, ECMO carries significant risks, including mortality rates of 30%-50% and complications such as multiple organ failure, bleeding, thrombosis, and nerve damage.^2^^,^^5^^,^^6^ Advances in ECMO technology and management over the past 2 decades have improved survival. However, survivors often experience persistent sequelae, including physical, psychological, and functional impairments that diminish health-related quality of life and limit the recovery of preadmission function.^7^^,^^8^ These challenges create ongoing demand for inpatient and outpatient care, further straining healthcare systems under increasing pressure.^7^, ^8^, ^9^

ECMO is also one of the most expensive healthcare interventions, with substantial costs during the index hospitalization compounded by the ongoing healthcare needs of survivors. For example, in Australia, annual expenditure exceeds US$1.39 billion (2023), excluding additional post-discharge care costs.^10^ Despite these escalating costs, global ECMO use has risen dramatically, with 157,993 adult ECMO runs in the past decade (2014-2023), representing a 364% increase compared with the previous decade (2004-2013).^11^ The COVID-19 pandemic created unprecedented demand for ECMO, particularly venovenous ECMO (VV-ECMO). However, long-term registry data from the Extracorporeal Life Support Organisation (ELSO) show a steady rise in ECMO use since 2008.^12^ This trend coincides with prior respiratory pandemics, such as H1N1 influenza, and the **C**onventional Ventilatory Support Versus **E**xtracorporeal Membrane Oxygenation for **S**evere **A**dult **R**espiratory Failure (CESAR) trial,^13^ which demonstrated a survival benefit after referral to an ECMO center, and contributed to the expansion of these centers.^14^ This growing demand, together with ECMO’s clinical complexity, warrants a broader investigation into its long-term resource and economic burden.

Most existing studies have focused on healthcare resource utilization and costs during the index admission, leaving post-discharge outcomes and associated costs relatively underexplored. A more thorough understanding of the survivorship trajectory could help identify modifiable risk factors linked to poorer long-term outcomes and guide future policy to optimize service delivery and resource allocation.^15^ To address this, we performed a systematic review of the literature on post-discharge health resource utilization and costs for survivors of ECMO.

## Methods

A systematic search was conducted in Ovid MEDLINE, Ovid Embase, Ovid Emcare, Scopus, and Web of Science databases from inception to October 1, 2024, focusing on English-language studies reporting healthcare resource utilization and costs after ECMO. The review was prospectively registered with PROSPERO (CRD42024523437) and performed according to the **P**referred **R**eporting **I**tems for **S**ystematic Reviews and **M**eta-**A**nalysis (PRISMA) 2020 guidelines (Supplemental Table S1).^16^

Our search strategy incorporated subject headings and free-text terms covering: 1) “extracorporeal membrane oxygenation,” 2) “healthcare resource utilization,” and 3) “healthcare resource costs.” Searches were tailored to each database (Supplemental Table S2). Hand-searching and reference checking supplemented database searches to identify additional relevant studies.

### Study selection

We included studies that reported healthcare resource utilization and/or costs for adult (age ≥ 18 years) patients who received ECMO for any indication (venoarterial [VA], venovenous [VV], or extracorporeal cardiopulmonary resuscitation [ECPR]). Studies that included ECMO and non-ECMO patients, or pediatric and adult populations, were eligible only if specific data for adult ECMO patients were presented separately. We excluded studies reporting exclusively on healthcare resource utilization and/or costs during the index hospitalization. Two reviewers independently screened titles, abstracts, and full-text studies for eligibility based on predefined criteria (Table 1), with disagreements resolved by a third reviewer.

**Table 1:** Inclusion and exclusion criteria

Inclusion criteria	Exclusion criteria
1. Adult patients ≥ 18 years of age who received ECMO	1. Patients did not receive ECMO
2. Studies that include healthcare resource use and/or costs following ECMO support	2. Focuses specifically on the index hospitalization during which ECMO was provided
3. Primary studies	
	3. Adult and paediatric populations reported together, with results for adult ECMO patients not reported separately
	4. Available only in abstract form
	5. Non-English studies
	6. Systematic reviews, opinion pieces, and case studies

ECMO, extracorporeal membrane oxygenation.

### Data extraction

Two reviewers independently extracted data using Covidence software and a predefined data extraction form (Supplemental Appendix S1). The information extracted included the author, year of publication, country, study design, number of sites (single-center or multicenter), ECMO mode (VA, VV, or ECPR), ECMO indication, and healthcare resource utilization and costs. A third reviewer resolved any disagreements.

### Quality assessment

The quality of included studies was assessed using a tool specifically developed for costing studies, based on recommendations from the Consolidated Health Economic Evaluation Reporting Standards, the Drummond Checklist for economic evaluations, and relevant governmental guidelines (Supplemental Appendix S2).^17^ Formal validation studies have not yet been conducted; however, the tool offers a structured approach based on established recommendations. Each study received 1 point for reporting an item and an additional point if the item was deemed “appropriate.” Results were tabulated to derive a composite quality score, with a maximum possible score of 57 points. Higher scores indicated better study quality and lower risk of bias.

### Data synthesis

Resource utilization and cost data were summarized in tabular form (Table 2 and Supplemental Table S4). Healthcare resource utilization and/or costs measured at similar timepoints (within 30 days, 6 months, and 1 year) were synthesized and presented as mean and standard deviation (SD) or median and interquartile range (IQR), when available. Data were further categorized by ECMO mode and/or geographic region.

**Table 2:** Summary of included studies

First author (year)	Country	Study design	Study setting	ECMO mode	Sample size	Readmission rate [SD]	Quality assessment score
Bak (2024)	KR	RCS	NR	VA	217	6 mo: 41 of 217 (18.9%)	22 of 23 (95.7%)
Banning (2023)	UK, ES, DE, NO, LV, BE	RCT	NR	VA	12	1 y: 1 of 12 (8.3%)	23 of 25 (92.0%)
Briasoulis (2023)	US	RCS	NR	NR	753	30 d: 130 of 753 (17.2%), 90 d: 268 of 753 (35.6%)	27 of 36 (75.0%)
Chan (2024)	TW	RCS	NR	VA, VV	395	> 90 d after ECMO insertion: 227 of 395 (57.5%)	21 of 21 (100.0%)
Chen (2017)	TW	RCS	Metro, regional	NR	1137	NR	37 of 46 (80.4%)
Christian-Miller (2020)	US	RCS	Metro, rural	NR	23,876	30 d: 2296 of 22,907 (10.0%)	24 of 35 (68.6%)
Delnoij (2024)	NL	EE	NR	ECPR	70	NR	45 of 47 (95.7%)
Desch (2024)	DE, SI	RCT	NR	VA	209	1 y: 16 of 90 (17.8%)	22 of 26 (84.6%)
Duraes-Campos (2024)	ES, PT	RCS	NR	VA	34	29 (12-48) mo: 7 of 34 (20.6%)	16 of 19 (84.2%)
Fernando (2019)	CA	RCS	Metro, rural	NR	692	30 d: 67 of 415 (16.1%), 90 d: 125 of 415 (30.1%), 1 y: 208 of 415 (50.1%)	40 of 46 (87.0%)
Hess (2021)	US	RCS	NR	NR	115	NR	20 of 23 (87.0%)
Huesch (2018)	US	RCS	NR	NR	2948	30 d: 1291 of 2948 (43.8% [49.6%]); 1 y: 1786 of 2948 (60.6% [48.9%])	18 of 21 (85.6%)
Jaamaa-Holmberg (2020)	FI	RCS	Metro	VA, ECPR	102	NR	35 of 40 (87.5%)
Kim (2020)	KR	RCS	Metro, rural	NR	3826	Within 1 y: 32 of 3826 (0.8%)	34 of 40 (85.0%)
Mayer (2022)	US	RCS	NR	VA, VV, other: hybrid (VA-VV)	315	30 d: 21 of 315 (13.0%)∗	21 of 23 (91.3%)
Nuqali (2022)	US	RCS	NR	NR	10,723	30 d: 694 of 4229 (16.4%)	21 of 23 (91.3%)
Oh (2022)	KR	RCS	Metro, rural	VA, VV	18,697	NR	41 of 43 (95.3%)
Oude Lansink-Hartgring (2023)	NL	PCS, EE	NR	VA, VV, ECPR	428	NR	49 of 49 (100%)
Peek (2010)	UK	RCT, EE	Metro	VV	68	NR	55 of 56 (98.2%)
Sanaiha (2019)	US	RCS	Metro, regional, rural	NR	18,748	90 d: 3956 of 18,748 (21.1%)	36 of 43 (83.7%)
Scotti (2015)	US	RCS	NR	NR	132	30 d: 45 of 132 (34.1%), 90 d: 70 of 132 (53.0%)	36 of 43 (83.7%)
Tashtish (2020)	US	RCS	NR	VA	1641	30 d: 158 of 661 (23.9%)	30 of 42 (71.4%)
Varvoutis (2023)	US	RCS	Metro, regional, rural	NR	8317†	NR	44 of 49 (89.8%)
Vetrovec (2023)	US	RCS	NR	NR	338	45 d: PI stays: 67 of 338 (55.4%); 45 d: post-SNF: 38 of 338 (31.4%)	45 of 51 (88.2%)

BE, Belgium; CA, Canada; d, days; DE, Germany; ECPR, extracorporeal cardiopulmonary resuscitation; EE, economic evaluation; ES, Spain; FI, Finland; KR, Republic of Korea; LV, Latvia; mo, months; NL, The Netherlands; NO, Norway; NR, not reported; PCS, prospective cohort study; PI, postindex; PT, Portugal; RCS, retrospective cohort study; RCT, randomized controlled trial; SI, Slovenia; SNF, skilled nursing facility admissions; TW, Taiwan; UK, United Kingdom; US, United States; VA, venoarterial; VV, venovenous; y, years.

∗ Calculated from survivors.

† Weighted.

Proportions were pooled using a mixed logistic regression model with a random intercept to account for study-level variability, with τ^2^ variance estimated via maximum likelihood estimation (Supplemental Table S5).

Costs reported in various currencies were converted to US dollars using the Purchasing Power Parity method, ensuring accurate cost comparisons by accounting for relative differences in purchasing power.^18^ All costs were subsequently adjusted for inflation using the Consumer Price Index to reflect rates in 2023 US dollars (Supplemental Table S4).^19^

## Results

**Figure 1:**
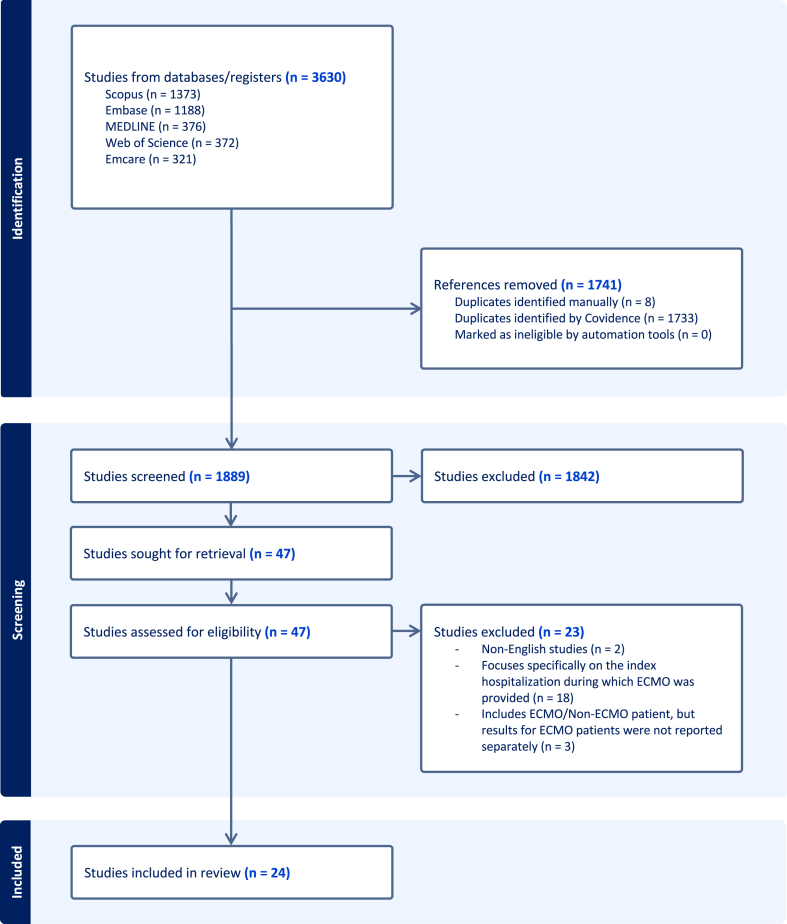
PRISMA flow diagram shows the number of studies included and excluded at each stage of screening and full-text review. PRISMA, Preferred Reporting Items for Systematic Reviews and Meta-Analysis.

Our search identified 1889 articles, of which 24 studies met the inclusion criteria (Fig. 1). Of these, 12 studies were conducted in North America (12 of 24, 50.0%),^20^, ^21^, ^22^, ^23^, ^24^, ^25^, ^26^, ^27^, ^28^, ^29^, ^30^, ^31^ 5 in Asia (5 of 24, 20.8%),^32^, ^33^, ^34^, ^35^ and 7 in Europe (7 of 24, 29.2%) (Table 3).^36^, ^37^, ^38^, ^39^, ^40^, ^41^, ^42^ Sample sizes ranged from 12 to 23,876 patients, with publication years from 2010 to 2024. Most studies (18 of 24, 75.0%) were published in the past 5 years.^20^^,^^21^^,^^23^^,^^25^^,^^26^^,^^29^, ^30^, ^31^, ^32^^,^^34^, ^35^, ^36^, ^37^, ^38^, ^39^, ^40^, ^41^^,^^43^ The majority of studies were retrospective cohort studies (19 of 24, 79.2%),^20^, ^21^, ^22^, ^23^, ^24^, ^25^, ^26^, ^27^, ^28^, ^29^, ^30^, ^31^, ^32^, ^33^, ^34^, ^35^^,^^39^^,^^40^^,^^43^ whereas 5 (5 of 24, 20.8%) were randomized controlled trials or economic evaluations that collected data on healthcare use and costs.^36^, ^37^, ^38^^,^^41^^,^^42^

**Table 3:** Cohort demographics (N = 24)

Characteristics	
Geographic region	
North America	12 (50.0%)
Asia	5 (20.8%)
Europe	7 (29.2%)
Study type	
Cohort	20 (83.3%)
Randomized controlled trial	3 (12.5%)
Economic evaluation	3 (12.5%)
Study scope	
Multicenter	17 (70.8%)
Single center	2 (8.3%)
Not reported	5 (20.8%)
Study population	
ECMO/ECLS diagnosis/procedure code	9 (37.5%)
Cardiogenic shock	9 (37.5%)
Other cardiac indication	5 (20.8%)
Respiratory failure	1 (4.2%)
ECMO mode	
VA	10 (43.5%)
VV	5 (20.8%)
ECPR	3 (12.5%)
Other	1 (0.04%)
Not reported	12 (50.0%)
Timepoints measured∗	
30 days	11 (45.8%)
45-90 days	6 (25.0%)
6 months	4 (16.7%)
1 year	12 (50.0%)
>1 year	5 (20.8%)

ECLS, Extracorporeal Life Support Organisation; ECMO, extracorporeal membrane oxygenation; ECPR, extracorporeal cardiopulmonary resuscitation; VA, venoarterial; VV, venovenous.

∗ Studies could measure readmissions at multiple time-points.

Most studies (11 of 24, 57.9%) adopted a healthcare payer perspective,^20^, ^21^, ^22^^,^^27^, ^28^, ^29^^,^^31^^,^^33^, ^34^, ^35^^,^^42^ followed by hospital (7 of 24, 29.2%)^20^^,^^27^^,^^29^^,^^30^^,^^35^^,^^41^^,^^42^ and societal perspectives (3 of 24, 12.5%).^37^^,^^41^^,^^42^ Some studies reported costs from more than one perspective. The overall quality of studies was generally high, with consistent reporting of funding sources and conflicts of interest. Key areas where reporting was inadequate included costing methodology, sample size calculation, resource identification, cost classification, and resource evaluation, indicating that these aspects were often insufficiently detailed. Complete quality assessments and scores are presented in Supplemental Table S6.

In-hospital mortality rates were reported in 18 of the 24 studies (75%), ranging from 20.6% to 67.4%.^20^, ^21^, ^22^^,^^24^, ^25^, ^26^, ^27^, ^28^, ^29^, ^30^, ^31^^,^^33^^,^^36^^,^^38^, ^39^, ^40^, ^41^^,^^43^ The combined proportion of hospital mortality was 47% (95% confidence interval 0.42-0.53), with a prediction interval of 0.24-0.71.

Among survivors of ECMO, readmissions were common, with 8 of the 24 studies (33.3%) reporting 30-day readmission rates, ranging between 10.0% and 43.8%.^20^, ^21^, ^22^^,^^24^, ^25^, ^26^^,^^28^^,^^29^ Long-term readmission rates were reported in 12 of the 24 studies (50.0%), with post-discharge periods from 45 days to 1 year.^20^^,^^22^^,^^24^^,^^27^^,^^28^^,^^31^^,^^32^^,^^34^^,^^36^^,^^38^^,^^39^^,^^43^ Reported readmission rates included 55.4% at 45 days,^31^ 21.1%-57.5% at 90 days,^27^^,^^32^ 18.9% at 6 months,^43^ and 0.8%-60.6% at 1 year.^24^^,^^34^ One study reported a 12- to 48-month readmission rate of 20.6%.^39^

Readmission length of stay (LOS) was reported in 5 of the 24 studies (20.8%), with mean LOS ranging from 10 to 51 days,^27^, ^28^, ^29^^,^^31^ and median LOS reported as 7^26^ and 38 days.^29^ Mortality rates among 30-day readmissions were reported in 2 studies (7.4%^29^ and 9.7%^26^), whereas a third study reported a 90-day readmission mortality rate of 5.4%.^27^

The mode of ECMO was often unspecified and few studies^22^^,^^33^^,^^39^^,^^41^ (4 of 24, 16.7%) explicitly stated whether they excluded cases where ECMO was used intraoperatively but discontinued postoperatively (eg, transplant, cardiac surgery). Of the 24 studies, 7 (29.2%) reported data on VA ECMO,^25^^,^^29^^,^^35^^,^^38^, ^39^, ^40^, ^41^ 5 (20.8%) on VV ECMO,^25^^,^^32^^,^^35^^,^^41^^,^^42^ 1 on a hybrid form of VA-VV ECMO,^25^ and 3 on ECPR.^37^^,^^40^^,^^41^

Hospital readmissions were identified as the primary indicator of healthcare resource utilization among ECMO patients. Of the 24 included studies, 3 (12.5%) provided data on readmission costs at 30, 45, and 90 days post-discharge.^27^, ^28^, ^29^ The mean and median 30-day readmission costs reported across the studies in our review were $62,150^28^ and $92,316,^29^ respectively. For 90-day readmissions, mean costs were $40,881^27^ and $43,445.^28^ One study reported 1-year total readmission costs for 2 ECPR patients, with a mean of $4458.^37^

Five of the 24 studies (20.8%) reported total healthcare costs from the index admission to 6 months or 1 year.^22^^,^^29^^,^^31^^,^^35^^,^^41^^,^^42^ At 6 months, 1 study reported a mean total cost of $162,564,^42^ whereas, at 1 year, reported mean costs were $190,169 (SD $178,556)^22^ and $243,747 (SD $252,181).^41^ Median costs over the same period were reported in 2 of the 24 (8.3%) studies, with costs of $58,791 (IQR $32,662-$110,356)^45^ at 6 months and $136,563 (IQR $61,498-$252,476)^22^ at 1 year.

Beyond readmission costs, 13 of the 24 (54.2%) studies reported additional costs for emergency department visits, complex continuing care, long-term care, rehabilitation, and home care.^21^^,^^22^^,^^27^^,^^29^, ^30^, ^31^^,^^33^, ^34^, ^35^^,^^37^^,^^40^, ^41^, ^42^ Other cost categories included outpatient services, laboratory tests, pharmaceuticals, patient self-payment, insurance coverage, absenteeism, and follow-up care over 1 year.

Only 4 studies^37^^,^^40^, ^41^, ^42^ (4 of 24, 16.7%) measured health economic outcomes as cost per quality-adjusted life-year (QALY), incorporating mortality and quality-of-life metrics. Two studies^41^^,^^42^ (2 of 24, 8.3%) reported cost per QALY for ECMO patients, and 2 (2 of 24, 8.3%)^37^^,^^40^ reported cost per QALY gained for ECPR patients (Supplemental Table S4). Three^40^, ^41^, ^42^ of the 4 studies reported incremental cost-effectiveness ratios that fall below commonly accepted willingness-to-pay thresholds, indicating that ECMO in the scenarios assessed may be cost-effective.

## Discussion

This systematic review provides important insights into mortality outcomes, post-discharge health resource utilization, and the financial burden associated with ECMO survivorship. Although ECMO is a lifesaving intervention for critically ill patients, its long-term implications for patient outcomes and healthcare costs are profound.^2^ Our review identified 24 studies, primarily from North America, mostly published in the last 5 years. This growing body of evidence reflects the increasing use of ECMO and a rising interest in studies reporting post-discharge outcomes. High readmission rates were observed, ranging from 10.0% to 43.8% at 30 days and persisting up to 20.6% from 12 to 48 months. The associated costs were also substantial, with mean post-discharge costs of $62,150 at 30 days and cumulative 1-year costs totaling $243,747.

Many survivors of ECMO require additional support after discharge to manage ongoing health challenges.^7^, ^8^, ^9^ Our review has revealed considerable variation in readmission rates, ranging from 10.0% to 43.8%, compared with 16.9% reported for general intensive care unit (ICU) survivors.^9^ Accordingly, these higher rates contribute to more frequent and costly readmissions, with 1-year post-discharge costs up to 22-fold higher than those for general ICU survivors. At 30 days, readmission costs for ECMO patients were as high as $62,150, with costs ranging from $40,881 to $43,445 at 90 days, compared with $11,408 for general ICU patients at 1 year.^44^ Lower readmission rates among general ICU survivors correspond to reduced overall costs despite similar reasons for readmission linked to the index admission diagnosis.^45^ However, it remains unclear whether these higher readmission rates and costs are directly attributable to ECMO itself, illness severity, or psychological and emotional factors that influence future healthcare use.^46^ For ECMO patients initially treated for cardiogenic shock, procedure-related complications, and severe baseline comorbidities frequently drive readmissions.^47^, ^48^, ^49^ Factors influencing readmission rates can vary depending on the patient’s underlying condition and indication for ECMO. This suggests the need for additional comparative studies with non-ECMO patients of similar illness severity to better understand the key drivers of extended healthcare needs. Targeted rehabilitation programs, improved community-based care referrals, follow-up clinics, and education for primary care providers could reduce unplanned readmissions and costs while also addressing post-ICU impairments.^7^^,^^9^

Despite improved survival rates, long-term mortality after ECMO remains high. In our review, 1-year mortality rates among survivors of ECMO ranged from 35.3% to 82.2%, compared with 7%-21% in general ICU cohorts.^44^ However, for many patients declined ECMO, survival outcomes may be even poorer. We did not identify any studies comparing survival or resource use between ECMO patients and those declined for ECMO, making it difficult to fully assess the intervention’s effectiveness. Improving patient selection through individualized prognostication, ensuring ECMO is reserved for patients most likely to benefit, presents a key opportunity to reduce healthcare strain and costs and improve overall patient outcomes.^8^^,^^50^ Further research on patient-centered outcomes, such as QALYs, and economic outcomes, such as incremental cost-effectiveness ratios, is needed to understand whether the benefits of ECMO, including reduced mortality, justify post-discharge resource use and long-term costs.

When evaluating ECMO-associated costs, both hospital and societal perspectives must be considered. Beyond direct healthcare costs incurred by the hospital, understanding the full economic impact of ECMO on patients and their families requires consideration of patient-centered factors, such as lost productivity and time spent attending medical appointments. Future research should explore the impact of rapid discharge on caregiver burden, as it may increase the demand for post-discharge support services and shift the cost burden to patients or primary care providers. Adopting a broader cost perspective over an extended time horizon would provide a clearer understanding of ECMO’s full-cost spectrum, strengthening the evidence base for this population.

A key strength of this review is its clearly defined research question and comprehensive search strategy, which involved screening 5 databases to capture a broad scope of studies. Independent data extraction by multiple reviewers using a standardized form further supports the reliability of our findings. However, limitations must be acknowledged.

There were very few studies to include in this review compared with the large number of ECMO studies published overall. The evidence base is limited by insufficient detail on factors such as illness severity, ECMO duration, organ failure, age, and pre-existing comorbidities, all of which may have influenced outcomes. Most studies did not clearly report intraoperative ECMO use or in what proportion of patients it was discontinued postoperatively. This should be reported more clearly in future studies.

Including studies with different ECMO modes (eg, ECPR, VA, and VV) and clinical trajectories (eg, cardiogenic shock, respiratory failure, and cardiac arrest) complicates direct comparisons. This was further limited by variability in follow-up duration (30 days to 1 year), as there are fewer data points available at specific intervals. In addition, most studies did not stratify outcomes by ECMO indication, limiting subgroup analyses and the ability to assess cost differences. Despite these limitations, their inclusion in this review is justified, as ongoing care needs, resource use, and cost patterns for ECMO survivors is likely to overlap across indications.

Most included studies were retrospective cohort studies, which, although generally well-designed, often lacked robust costing methods and sample size calculations. Some studies did not clearly report which resources were included in the cost estimates or how resource use was measured, limiting the quality of available evidence. One study presented costs graphically without precise numerical data.^33^ Attempts to contact the authors for clarification were unsuccessful, which may have limited the comprehensiveness of our review.

Differences in cost perspectives (hospital, societal, or healthcare payer), cost reporting, and outcome measures across studies posed additional challenges and complicated interpretations. Although we standardized costs to 2023 US dollars using the Consumer Price Index and Purchasing Power Parity, the heterogeneity of study designs precluded meta-analysis. Inconsistent data, such as missing SD or IQR data, also limited our ability to calculate weighted means based on the sample size.

There was limited information presented on health economic outcomes, such as cost per QALY. The absence of high-quality randomized controlled trial data–aside from the Peek et al study for ECMO patients and the Delnoij et al study for ECPR patients–limited the ability to assess ECMO’s cost-effectiveness. Additionally, comparing QALYs between ECMO patients and those declined ECMO is challenging due to baseline differences, and ongoing mortality further complicates interpretation. No studies directly compared outcomes or resource use between ECMO patients and those declined for ECMO.

Our analysis employed a mixed logistic regression model with a random intercept to pool proportions. However, the limited number of included studies and the heterogeneity among them may impact the generalizability of our findings. Also, the restriction to English-language studies could further limit generalizability, potentially underrepresenting ECMO outcomes from a broader global perspective.

Although ECMO use had already been rising before 2020,^12^ the COVID-19 pandemic likely accelerated this trend, especially for VV ECMO among critically ill COVID-19 patients. This may have skewed the distribution of studies in this review, as patient populations and clinical indications evolved during the pandemic. Future research should account for both pre-pandemic trends and the pandemic’s impact on ECMO survivorship, clearly distinguishing by ECMO mode, duration, and patient demographics.

A more detailed breakdown of costs, including both direct (eg, hospitalizations, specialized staff, equipment, medications) and indirect (eg, long-term rehabilitation, impact on employment and productivity) expenses, is needed. The included studies did not account for these costs, which affect not only patients and caregivers but also hospitals and health systems. Furthermore, current data have not kept pace with the growing demand for ECMO, indicating a need for more consistent and comprehensive cost analyses in future studies to guide healthcare resource allocation for ECMO survivorship.

## Conclusions

ECMO imposes considerable ongoing resource and financial demands on healthcare systems, patients, caregivers, and society. Our review has revealed very limited information on post-ECMO healthcare utilization, emphasizing the need for more comprehensive and granular data on ECMO survivorship. Understanding the full scope of ECMO-related costs, including equipment, specialized staff, length of stay, and post-discharge care across the different patient populations and clinical settings, is essential. These data are critical for assessing ECMO’s cost-effectiveness, improving patient selection, and informing service planning and resource allocation, particularly at the intersection between acute and community care.^15^
