# Supplementary material for: Health Resource Utilization and Outcomes Among Patients Who Receive Extracorporeal Membrane Oxygenation
Source: CJC Open. 2025 Mar 31;7(6):750–8. doi: 10.1016/j.cjco.2025.03.019 (PMC12198622; doi:10.1016/j.cjco.2025.03.019)
Supplement: Supplementary Material [file mmc1.docx]

**Supplemental Material**

**Article Name:** Health Resource Utilization and Outcomes Among Patients Who Receive ECMO: A Systematic Review

**Journal Name:** Canadian Journal of Cardiology Open (CJCO)

Vicki Papanikolaou, Ethan Goh, Alayna Carrandi, Anais Charles-Nelson, Kiran Kottakal, Lorena Romero, Carol L. Hodgson, Alisa M. Higgins

Table of Contents

[Supplemental Table S1. PRISMA 2020 Main Checklist 3](#_Toc193660238)

[Supplemental Table S2. Electronic Database Search Strategy 8](#_Toc193660239)

[Supplemental Table S3. Outcome Parameters for ECMO group 13](#_Toc193660240)

[Supplemental Table S4. Studies Reporting Costs or Charges of Healthcare Resource Use 17](#_Toc193660241)

[Supplemental Table S5. Summary Table of Pooled Proportions 24](#_Toc193660242)

[Supplemental Table S6. Quality Assessment of Included Studies 25](#_Toc193660243)

[Supplemental Appendix S1 - Pre-defined Data Extraction Form 43](#_Toc193660244)

[Supplemental Appendix S2 - Quality Assessment Template 47](#_Toc193660245)

# Supplemental Table S1. PRISMA 2020 Main Checklist

| **Topic** | **No.** | **Item** | **Location where item is reported** |
| --- | --- | --- | --- |
| **TITLE** |  |  |  |
| **Title** | 1 | Identify the report as a systematic review. | Title page |
| **ABSTRACT** |  |  |  |
| **Abstract** | 2 | See the PRISMA 2020 for Abstracts checklist | Page 3 |
| **INTRODUCTION** |  |  |  |
| **Rationale** | 3 | Describe the rationale for the review in the context of existing knowledge. | Pages 4-5 |
| **Objectives** | 4 | Provide an explicit statement of the objective(s) or question(s) the review addresses. | Page 5 |
| **METHODS** |  |  |  |
| **Eligibility criteria** | 5 | Specify the inclusion and exclusion criteria for the review and how studies were grouped for the synthesis. | Page 6, Table 1 |
| **Information sources** | 6 | Specify all databases, registers, websites, organisations, reference lists and other sources searched or consulted to identify studies. Specify the date when each source was last searched or consulted. | Pages 5-6 |
| **Search strategy** | 7 | Present the full search strategies for all databases, registers and websites, including any filters and limits used. | Supplemental Table S2 |
| **Selection process** | 8 | Specify the methods used to decide whether a study met the inclusion criteria of the review, including how many reviewers screened each record and each report retrieved, whether they worked independently, and if applicable, details of automation tools used in the process. | Page 6 |
| **Data collection process** | 9 | Specify the methods used to collect data from reports, including how many reviewers collected data from each report, whether they worked independently, any processes for obtaining or confirming data from study investigators, and if applicable, details of automation tools used in the process. | Page 6, Supplemental Appendix S1 |
| **Data items** | 10a | List and define all outcomes for which data were sought. Specify whether all results that were compatible with each outcome domain in each study were sought (e.g. for all measures, time points, analyses), and if not, the methods used to decide which results to collect. | Page 7 |
|  | 10b | List and define all other variables for which data were sought (e.g. participant and intervention characteristics, funding sources). Describe any assumptions made about any missing or unclear information. | Page 7 |
| **Study risk of bias assessment** | 11 | Specify the methods used to assess risk of bias in the included studies, including details of the tool(s) used, how many reviewers assessed each study and whether they worked independently, and if applicable, details of automation tools used in the process. | Page 6, Supplemental Appendix S2 |
| **Effect measures** | 12 | Specify for each outcome the effect measure(s) (e.g. risk ratio, mean difference) used in the synthesis or presentation of results. | Page 7 |
| **Synthesis methods** | 13a | Describe the processes used to decide which studies were eligible for each synthesis (e.g. tabulating the study intervention characteristics and comparing against the planned groups for each synthesis (item 5)). | Page 7 |
|  | 13b | Describe any methods required to prepare the data for presentation or synthesis, such as handling of missing summary statistics, or data conversions. | Page 7 |
|  | 13c | Describe any methods used to tabulate or visually display results of individual studies and synthesis. | Page 7 |
|  | 13d | Describe any methods used to synthesize results and provide a rationale for the choice(s). If meta-analysis was performed, describe the model(s), method(s) to identify the presence and extent of statistical heterogeneity, and software package(s) used. | Page 7 |
|  | 13e | Describe any methods used to explore possible causes of heterogeneity among study results (e.g. subgroup analysis, meta-regression). | Page 7 |
|  | 13f | Describe any sensitivity analyses conducted to assess robustness of the synthesized results. | N/A |
| **Reporting bias assessment** | 14 | Describe any methods used to assess risk of bias due to missing results in a synthesis (arising from reporting biases). | N/A |
| **Certainty assessment** | 15 | Describe any methods used to assess certainty (or confidence) in the body of evidence for an outcome. | N/A |
| **RESULTS** |  |  |  |
| **Study selection** | 16a | Describe the results of the search and selection process, from the number of records identified in the search to the number of studies included in the review, ideally using a flow diagram. | Page 6 and Figure 1. |
|  | 16b | Cite studies that might appear to meet the inclusion criteria, but which were excluded, and explain why they were excluded. | N/A |
| **Study characteristics** | 17 | Cite each included study and present its characteristics. | Table 2 |
| **Risk of bias in studies** | 18 | Present assessments of risk of bias for each included study. | Supplemental Table S6 |
| **Results of individual studies** | 19 | For all outcomes, present, for each study: (a) summary statistics for each group (where appropriate) and (b) an effect estimates and its precision (e.g. confidence/credible interval), ideally using structured tables or plots. | Table 2, Supplemental Table S3 and S4 |
| **Results of synthesis** | 20a | For each synthesis, briefly summarise the characteristics and risk of bias among contributing studies. | N/A |
|  | 20b | Present results of all statistical synthesis conducted. If meta-analysis was done, present for each the summary estimate and its precision (e.g. confidence/credible interval) and measures of statistical heterogeneity. If comparing groups, describe the direction of the effect. | N/A |
|  | 20c | Present results of all investigations of possible causes of heterogeneity among study results. | N/A |
|  | 20d | Present results of all sensitivity analyses conducted to assess the robustness of the synthesized results. | N/A |
| **Reporting biases** | 21 | Present assessments of risk of bias due to missing results (arising from reporting biases) for each synthesis assessed. | Supplemental Table S6 |
| **Certainty of evidence** | 22 | Present assessments of certainty (or confidence) in the body of evidence for each outcome assessed. | Supplemental Table S3 and S4 |
| **DISCUSSION** |  |  |  |
| **Discussion** | 23a | Provide a general interpretation of the results in the context of other evidence. | Pages 10-15 |
|  | 23b | Discuss any limitations of the evidence included in the review. | Pages 12-15 |
|  | 23c | Discuss any limitations of the review processes used. | Pages 12-15 |
|  | 23d | Discuss implications of the results for practice, policy, and future research. | Pages 12-15 |
| **OTHER INFORMATION** |  |  |  |
| **Registration and protocol** | 24a | Provide registration information for the review, including register name and registration number, or state that the review was not registered. | Page 5 |
|  | 24b | Indicate where the review protocol can be accessed, or state that a protocol was not prepared. | N/A |
|  | 24c | Describe and explain any amendments to information provided at registration or in the protocol. | N/A |
| **Support** | 25 | Describe sources of financial or non-financial support for the review, and the role of the funders or sponsors in the review. | Tile page |
| **Competing interests** | 26 | Declare any competing interests of review authors. | Title page |
| **Availability of data, code and other materials** | 27 | Report which of the following are publicly available and where they can be found template data collection forms; data extracted from included studies; data used for all analyses; analytic code; any other materials used in the review. | Supplemental Material |

*From:*  Page MJ, McKenzie JE, Bossuyt PM, Boutron I, Hoffmann TC, Mulrow CD, et al. The PRISMA 2020 statement: an updated guideline for reporting systematic reviews. BMJ 2021;372:n71. doi: 10.1136/bmj.n71. This work is licensed under CC BY 4.0. To view a copy of this license, visit <https://creativecommons.org/licenses/by/4.0/>

# Supplemental Table S2. Electronic Database Search Strategy

| **Ovid MEDLINE(R) and Epub Ahead of Print, In-Process, In-Data-Review & Other Non-Indexed Citations, Daily and Versions <1946 to October 1, 2024>** | |
| --- | --- |
| 1 | Extracorporeal Membrane Oxygenation/ |
| 2 | Oxygenators, Membrane/ |
| 3 | Extracorporeal Circulation/ |
| 4 | (ECMO or ECPR or ECLS or VAECMO or VVECMO or VVECLS or mECMO).mp. |
| 5 | membran* oxygenat*.mp. |
| 6 | ((veno-venous or venovenous or veno-arterial or venoarterial or arterio-venous or VV or VA) adj (extracorp* or extra-corp*)).mp. |
| 7 | ((extracorp* or extra-corp*) and (oxygen* or membran*)).mp. |
| 8 | ((extracorp* or extra-corp*) adj (cardiopulmonary resuscitation or CPR or lung* or circulat* or blood gas)).mp. |
| 9 | extrapulmonary oxygenation.mp. |
| 10 | ((extracorp* or extra-corp*) and (gas transfer or gas flow or gas exchange or oxygen transfer or O2 transfer)).mp. |
| 11 | ((extracorp* or extra-corp*) and respiratory assist*).mp. |
| 12 | ((extracorp* or extra-corp*) and (life support or lung support or respiratory support)).mp. |
| 13 | or/1-12 |
| 14 | Patient Readmission/ |
| 15 | Health Care Costs/ |
| 16 | Hospital Costs/ |
| 17 | Rehabilitation/ |
| 18 | (re-admission* or readmission* or readmit* or re-admit*).ti,ab,kw. |
| 19 | (rehospitali?ation* or re-hospitali?ation* or rehospitali?ed or re-hospitali?ed or rehospitali?ing or re-hospitali?ing).ti,ab,kw. |
| 20 | (Hospital cost? or health facility cost? or Hospitali?ation cost? or Hospital financ* or Hospital charge?).ti,ab,kw. |
| 21 | ((health care or healthcare or medical care or treatment) adj cost?).ti,ab,kw. |
| 22 | (hospital expenditure? or hospital expense?).ti,ab,kw. |
| 23 | ((health care or healthcare or health service* or health resource*) adj3 ("reusing" or "re-using" or "re-used" or "reused" or "re-us?age" or "reus?age")).ti,ab,kw. |
| 24 | ((health care or healthcare or health service* or health resource*) adj3 ("use" or utili?ation* or "us?age" or "re-use" or "reuse" or re-utili?ation* or reutili?ation* or cost*)).ti,ab,kw. |
| 25 | ((hospital or critical care or intensive care or emergency department* or emergency hospital service* or hospital emergency service* or emergency outpatient unit* or emergency room* or emergency unit* or emergency ward* or emergency outpatient unit*) adj2 ("use" or utili?ation* or "us?age" or "re-use" or "reuse" or re-utili?ation* or reutili?ation*)).ti,ab,kw. |
| 26 | (repeat* adj2 (hospitali* or admission* or admitted)).ti,ab,kw. |
| 27 | (rehabilitation care or heart rehabilitation or pulmonary rehabilitation).ti,ab,kw. |
| 28 | or/14-27 |
| 29 | 13 and 28 |
| 30 | exp animals/ not humans.sh. |
| 31 | 29 not 30 |
| 32 | (exp child/ or exp infant/) not exp adult/ |
| 33 | 31 not 32 |
| 34 | limit 33 to (autobiography or bibliography or biography or case reports or clinical trial, veterinary or comment or congress or dictionary or directory or editorial or interview or legal case or letter or news or newspaper article or observational study, veterinary or patient education handout or personal narrative or randomized controlled trial, veterinary or webcast) |
| 35 | 33 not 34 |
| 36 | limit 35 to ("review" or "scientific integrity review" or "systematic review") |
| 37 | 35 not 36 |
| 38 | limit 37 to meta analysis |
| 39 | 37 not 38 |
| 40 | Extracorporeal Membrane Oxygenation/ec |
| 41 | 40 not 32 |
| 42 | limit 41 to (autobiography or bibliography or biography or case reports or clinical trial, veterinary or comment or congress or dictionary or directory or editorial or interview or legal case or letter or news or newspaper article or observational study, veterinary or patient education handout or personal narrative or randomized controlled trial, veterinary or webcast) |
| 43 | 41 not 42 |
| 44 | limit 43 to ("review" or "scientific integrity review" or "systematic review") |
| 45 | 43 not 44 |
| 46 | limit 45 to meta analysis |
| 47 | 45 not 46 |
| 48 | 39 or 47 |
| **Embase Classic+Embase <1947 to 2024 October 1> and Ovid Emcare** | |
| 1 | extracorporeal circulation/ or extracorporeal oxygenation/ or arterio-venous ecmo/ or veno-arterial ecmo/ or veno-venous ecmo/ |
| 2 | extracorporeal membrane oxygenation device/ |
| 3 | membrane oxygenator/ |
| 4 | (ECMO or ECPR or ECLS or VAECMO or VVECMO or VVECLS or mECMO).mp. |
| 5 | membran* oxygenat*.mp. |
| 6 | ((veno-venous or venovenous or veno-arterial or venoarterial or arterio-venous or VV or VA) adj (extracorp* or extra-corp*)).mp. |
| 7 | ((extracorp* or extra-corp*) and (oxygen* or membran*)).mp. |
| 8 | ((extracorp* or extra-corp*) adj (cardiopulmonary resuscitation or CPR or lung* or circulat* or blood gas)).mp. |
| 9 | extrapulmonary oxygenation.mp. |
| 10 | ((extracorp* or extra-corp*) and (gas transfer or gas flow or gas exchange or oxygen transfer or O2 transfer)).mp. |
| 11 | ((extracorp* or extra-corp*) and respiratory assist*).mp. |
| 12 | ((extracorp* or extra-corp*) and (life support or lung support or respiratory support)).mp. |
| 13 | or/1-12 |
| 14 | Hospital readmission/ |
| 15 | Health care utilization/ |
| 16 | Health care cost/ or Hospital cost/ |
| 17 | Rehabilitation/ |
| 18 | (re-admission* or readmission* or readmit* or re-admit*).ti,ab,kw. |
| 19 | (rehospitali?ation* or re-hospitali?ation* or rehospitali?ed or re-hospitali?ed or rehospitali?ing or re-hospitali?ing).ti,ab,kw. |
| 20 | (Hospital cost? or health facility cost? or Hospitali?ation cost? or Hospital financ* or Hospital charge?).ti,ab,kw. |
| 21 | ((health care or healthcare or medical care or treatment) adj cost?).ti,ab,kw. |
| 22 | (hospital expenditure? or hospital expense?).ti,ab,kw. |
| 23 | ((health care or healthcare or health service* or health resource*) adj3 ("reusing" or "re-using" or "re-used" or "reused" or "re-us?age" or "reus?age")).ti,ab,kw. |
| 24 | ((health care or healthcare or health service* or health resource*) adj3 ("use" or utili?ation* or "us?age" or "re-use" or "reuse" or re-utili?ation* or reutili?ation* or cost*)).ti,ab,kw. |
| 25 | ((hospital or critical care or intensive care or emergency department* or emergency hospital service* or hospital emergency service* or emergency outpatient unit* or emergency room* or emergency unit* or emergency ward* or emergency outpatient unit*) adj2 ("use" or utili?ation* or "us?age" or "re-use" or "reuse" or re-utili?ation* or reutili?ation*)).ti,ab,kw. |
| 26 | (repeat* adj2 (hospitali* or admission* or admitted)).ti,ab,kw. |
| 27 | (rehabilitation care or heart rehabilitation or pulmonary rehabilitation).ti,ab,kw. |
| 28 | or/14-27 |
| 29 | 13 and 28 |
| 30 | (exp animal/ or exp invertebrate/ or animal.hw. or nonhuman/) not exp human/ |
| 31 | 29 not 30 |
| 32 | exp child/ not exp adult/ |
| 33 | 31 not 32 |
| 34 | limit 33 to (conference abstract or editorial or letter or note or short survey) |
| 35 | 33 not 34 |
| 36 | limit 35 to (meta analysis or "systematic review") |
| 37 | 35 not 36 |
| 38 | (autobiography or bibliography or biography or case report or veterinary or comment or dictionary or directory or editorial or interview or legal case or letter or news or newspaper or patient education or personal narrative or webcast).ti. |
| 39 | 37 not 38 |
| **Scopus** | |
| 1 | ( TITLE-ABS-KEY ( ( ecmo OR ecpr OR ecls OR vaecmo OR vvecmo OR vvecls OR mecmo ) ) OR TITLE-ABS-KEY ( ( ( "veno-venous" OR venovenous OR "veno-arterial" OR venoarterial OR "arterio-venous" OR vv OR va ) W/0 ( extracorp* OR "extra-corp*" ) ) ) OR TITLE-ABS-KEY ( ( ( extracorp* OR "extra-corp*" ) AND ( oxygen* OR membran* OR "gas transfer" OR "gas flow" OR "gas exchange" OR "oxygen transfer" OR "o2 transfer" ) ) ) OR TITLE-ABS-KEY ( ( ( extracorp* OR "extra-corp*" ) W/0 ( "cardiopulmonary resuscitation" OR cpr OR lung* OR circulat* OR "blood gas" ) ) ) OR TITLE-ABS-KEY ( ( "membran* oxygenat*" OR "extrapulmonary oxygenation" ) ) OR TITLE-ABS-KEY ( ( ( extracorp* OR "extra-corp*" ) AND ( "respiratory assist*" OR "life support" OR "lung support" OR "respiratory support" ) ) ) ) |
| 2 | ( TITLE-ABS-KEY ( ( "re-admission*" OR readmission* OR readmit* OR "re-admit*" ) ) OR TITLE-ABS-KEY ( ( rehospitali?ation* OR "re-hospitali?ation*" OR rehospitali?ed OR "re-hospitali?ed" OR rehospitali?ing OR "re-hospitali?ing" ) ) OR TITLE-ABS-KEY ( ( "hospital cost?" OR "health facility cost?" OR "hospitali?ation cost?" OR "hospital financ*" OR "hospital charge?" ) ) OR TITLE-ABS-KEY ( ( ( "health care" OR healthcare OR "medical care" OR treatment ) W/0 cost? ) ) OR TITLE-ABS-KEY ( ( "hospital expenditure?" OR "hospital expense?" ) ) OR TITLE-ABS-KEY ( ( ( "health care" OR healthcare OR "health service*" OR "health resource*" ) W/2 ( "reusing" OR "re-using" OR "re-used" OR "reused" OR "re-us?age" OR "reus?age" ) ) ) OR TITLE-ABS-KEY ( ( ( "health care" OR healthcare OR "health service*" OR "health resource*" ) W/2 ( "use" OR utili?ation* OR "us?age" OR "re-use" OR "reuse" OR "re-utili?ation*" OR reutili?ation* OR cost* ) ) ) OR TITLE-ABS-KEY ( ( repeat* W/1 ( hospitali* OR admission* OR admitted ) ) ) OR TITLE-ABS-KEY ( ( "rehabilitation care" OR "heart rehabilitation" OR "pulmonary rehabilitation" ) ) ) |
| 3 | 1 AND 2 |
| 4 | Excluded document types  Excluded Review  Excluded Editorial  Excluded Letter  Excluded Note  Excluded Book chapter  Excluded Book  Excluded Short survey |
| 5 | AND NOT  TITLE ( ( autobiography OR bibliography OR biography OR "case report" OR veterinary OR comment OR dictionary OR directory OR editorial OR interview OR "legal case" OR letter OR news OR newspaper OR "patient education" OR "personal narrative" OR webcast ) ) |
| **Web of Science** | |
| 1 | ( ecmo OR ecpr OR ecls OR vaecmo OR vvecmo OR vvecls OR mecmo ) (Topic) or (("veno-venous" OR venovenous OR "veno-arterial" OR venoarterial OR "arterio-venous" OR vv OR va ) NEAR/0 ( extracorp* OR "extra-corp*" )) (Topic) or ((extracorp* OR "extra-corp*" ) AND ( oxygen* OR membran* OR "gas transfer" OR "gas flow" OR "gas exchange" OR "oxygen transfer" OR "o2 transfer" )) (Topic) or ((extracorp* OR "extra-corp*" ) NEAR/0 ( "cardiopulmonary resuscitation" OR cpr OR lung* OR circulat* OR "blood gas" )) (Topic) or ("membran* oxygenat*" OR "extrapulmonary oxygenation") (Topic) or ((extracorp* OR "extra-corp*" ) AND ( "respiratory assist*" OR "life support" OR "lung support" OR "respiratory support" )) (Topic) |
| 2 | ("re-admission*" OR readmission* OR readmit* OR "re-admit*") (Topic) or (rehospitali?ation* OR "re-hospitali?ation*" OR rehospitali?ed OR "re-hospitali?ed" OR rehospitali?ing OR "re-hospitali?ing") (Topic) or ("hospital cost?" OR "health facility cost?" OR "hospitali?ation cost?" OR "hospital financ*" OR "hospital charge?") (Topic) or (("health care" OR healthcare OR "medical care" OR treatment ) NEAR/0 cost?) (Topic) or ("hospital expenditure?" OR "hospital expense?") (Topic) or (("health care" OR healthcare OR "health service*" OR "health resource*" ) NEAR/2 ( "reusing" OR "re-using" OR "re-used" OR "reused" OR "re-us?age" OR "reus?age")) (Topic) or (("health care" OR healthcare OR "health service*" OR "health resource*" ) NEAR/2 ( "use" OR utili?ation* OR "us?age" OR "re-use" OR "reuse" OR "re-utili?ation*" OR reutili?ation* OR cost* )) (Topic) or (repeat* NEAR/1 ( hospitali* OR admission* OR admitted)) (Topic) or ("rehabilitation care" OR "heart rehabilitation" OR "pulmonary rehabilitation") (Topic) |
| 3 | 1 AND 2 |
| 4 | NOT Document Types: Review Article or Meeting Abstract or Book Chapters or Editorial Material or Letter |
| 5 | NOT (autobiography OR bibliography OR biography OR "case report" OR veterinary OR comment OR dictionary OR directory OR editorial OR interview OR "legal case" OR letter OR news OR newspaper OR "patient education" OR "personal narrative" OR webcast) (Title) |

# Supplemental Table S3. Outcome Parameters for ECMO group

| **Author** (year) | **ECMO duration** (days) | **In-hospital mortality** (n/N (%)) | **Readmission Time point** | **Readmission reason(s)** | **Readmission LOS** (days) | **Readmission mortality** (n/N (%)) | **Other mortality** (n/N (%)) | **Follow-up period** |
| --- | --- | --- | --- | --- | --- | --- | --- | --- |
| Bak (2024) | Early venting group: Median: 6 [IQR 4-8]  No or delayed venting: Median: 7 [IQR 5-12] | 66/217 (30.4%) | 6m | Arrhythmia or heart failure (excluding routine biopsy) | NR | NR | NR | 6m |
| Banning (2023) | NR | 7/12 (58.3%) | 1y | Heart failure | NR | NR | 30d: 7/12 (58.3%);  1y: 9/12 (75.0%) | 30d; 1y |
| Briasoulis (2023) | NR | 215/753 (28.5%) | 30d; 90d | Arrythmias/ pericardial diseases | NR | NR | NR | 30d; 90d |
| Chan (2024) | Median: 5 [IQR 3-8] | NR | >90d after ECMO insertion | Sepsis and infection | NR | NR | >90d after ECMO insertion: 62/395 (15.7%) | 20 [IQR: 8-56]; 34 [IQR: 15-68] |
| Chen (2017) | NR | 701/1137 (61.7%) | NR | NR | NR | NR | 1y: 863/1137 (75.9%, [95% CI, 73.4-78.4]) 5y: 936/1137 (82.3%, [95% CI, 79.3-85.3] 10y: 1027/1137 (90.3% [95% CI, 84.5-96.0]) | 1y; 2y; 3y; 4y; 5y |
| Christian-Miller (2020) | NR | 11516/22907 (50.3%) | 30d | NR | NR | NR | NR | 30d; 1y |
| Delnoij (2024) | NR | NR | 1y | NR | NR | NR | NR | 1y |
| Desch (2024) | Median: 2.7 [IQR 1.5-4.8] | 100/209 (48%) | 1y | NR | NR | NR | 1y: 115/209 (55.0%) | 1y |
| Duraes-Campos (2024) | Median: 10 [IQR 6-18] | 7/34 (20.6%) | 29(12-48)m | Heart failure | NR | NR | 29 (12-48)m: 4/34 (12%) | 29 (12-48)m |
| Fernando (2019) | NR | 277/692 (40%) | 30d; 90d; 1y | Electrical storm (2 cases) | NR | NR | 7d: 176/692 (25.4%) 30d: 252/692 (36.4%) 1y: 312/692 (45.1%) 2y: 339/692 (49.0%) 5y: 397/692 (57.4%) | 30d; 90d; 1y |
| Hess (2021) | NR | NR | NR | NR | NR | NR | 1y: 74/115 (64.3%) 2y: 76/115 (66.1%) 3y: 89/115 (77.4%) 4y: 98/115 (85.2%) 5y: 105/115 (91.3%) | 1y; 2y; 3y; 4y; 5y |
| Huesch  (2018) | NR | 1524/2948 (51.7%) (50) | 30d; 1y | NR | NR | NR | NR | 30d; 1y |
| Jaamaa-Holmberg (2020) | Median: 6 [IQR 6] | 35/102 (34.3%) | NR | NR | NR | NR | 1y: 36/102 (35.3%) | 1y |
| Kim (2020) | NR | NR | 1y | NR | NR | NR | 30d: 2873/3826 (75.1%) 6m: 3117/3826 (81.5%) 1y: 3144/3826 (82.2%) | 30d; 6m; 1y |
| Mayer (2022) | Mean: 12.8 (SD 14.9) Median: 7 [IQR 5-16] | 156/315 (50.0%) | 30d | NR | NR | NR | NR | 30d |
| Nuqali (2022) | NR | 5602/10723 (52%) | 30d | Sepsis, acute heart failure exacerbation and critical illness myopathy/ neuropathy | Median: 7 | 30d: 67/694 (9.7%) | NR | 30d |
| Oh (2022) | Median: 3 [IQR 1-7] | NR | NR | NR | NR | NR | 1y: 12653/18697 (67.7%); 3y: 449/4255 (10.6%) | 1y; 3y |
| Oude Lansink-Hartgring  (2023) | Median: 4.0 [IQR 1.8-9.0] | 198/428 (46%) | NR | NR | NR | NR | 1y: 215/428 (50%) | 6m; 1y |
| Peek  (2010) | Median: 9.0 [IQR 6.0-16.0] | NR | NR | NR | NR | NR | 6m: 33/68 (48.5%) | 6m |
| Sanaiha  (2019) | NR | 9412/18748 (50.2%) | 90d | Cardiac and respiratory-related | Mean: 9.9 (SD 0.5) | 90d: 1012/18748 (5.4%) | NR | 90d |
| Scotti (2015) | NR | 89/132 (67.4%) | 30d; 90d | CS, coronary atherosclerosis or shortness of breath | 30d: Mean 17.5;  90d: Mean 20.5 | NR | NR | 30d; 90d |
| Tashtish  (2020) | NR | 907/1641 (55.3%) | 30d | CE, complications of medical/device care, infection, gastroenteric/liver complication | Mean: 50.8 (SD 45.6); Median: 38 [IQR 24–62] | 30d: 12/158 (7.4%) | NR | 30d |
| Varvoutis  (2023) | NR | 3244/8317 (39.0%)^*^ | NR | NR | NR | NR | NR | 30d; 60d |
| Vetrovec  (2021) | NR | 217/338 (64.2%) | 45d | NR | Mean: 11.3 | NR | 45d: 250/338 (74.0%) | 45d |

*CE,* cardiovascular events*; CS,* cardiogenic shock*; d,* days; *LOS,* length of stay; *m,* months; *NR,* not reported; *y,* year;
^*^Weighted population

# Supplemental Table S4. Studies Reporting Costs or Charges of Healthcare Resource Use

| **Author (year)** | **Patient Population** | **Cost/QALY** (2023 USD) | **Cost perspective** | **Readmission costs** (2023 USD) | **Total costs** (2023 USD) | **Other costs** (2023 USD) |
| --- | --- | --- | --- | --- | --- | --- |
| Bak (2024) | Fulminant myocarditis requiring VA-ECMO | NR | NR | NR | NR | NR |
| Banning (2023) | CS due to MI | NR | NR | - | - | NR |
| Briasoulis (2023) | CS due to ST-elevation MI (STEMI) | NR | H; HP | - | - | NR |
| Chan (2024) | ECMO between September 1, 2009, and December 31, 2018 | NR | NR | NR | NR | NR |
| Chen (2017) | Post-cardiotomy CS | NR | HP | - | - | Inpatient medical expenditure:  Median: $69,257 [IQR 51,198 – 94,631] |
| Christian-Miller (2020) | ECLS | NR | HP | - | - | Total hospital charges: Obese: Mean: $660,397 [95% CI, 599,132- 721,659] Non-obese: Mean: $881,839 [95% CI, 826,240- 937,437] |
| Delnoij (2024) | Refractory OHCA | *(ECPR group):*  $188,284 | S | 1-year total readmission costs (*for the 2 readmitted ECPR patients*): Mean: $4458 | NR | *(For all 70 patients in the ECPR cohort):* Total hospital costs index admission: Mean: $28,134 General practitioner: Mean: $17 AHP (incl. mental care): Mean: $172 Homecare formal/informal: Mean: $650 Medication: Mean: $110 ED/EMS: Mean: $77 Outpatient clinic visits and treatment: Mean: $1071 Total productivity costs: Mean: $4023 |
| Desch (2024) | CS related to acute MI (AMICS) | NR | NR | NR | NR | NR |
| Duraes-Campos (2024) | Drug-refractory ES without a reversible trigger | NR | NR | NR | NR | NR |
| Fernando (2019) | ECMO intervention code (excluding elective cardiac surgery) | NR | HP | - | 1 year total costs *(available for 550 patients)* Mean: $190,169 (SD 178,556 )  1 year total costs *(available for 550 patients):* Median: $136,563 [IQR 61,498 - 252,476] | Inpatient care: Mean: $144,097 (SD 152,350), Median: $95,680 [IQR 40,402-193,820] Emergency department: Mean: $679 (SD 936), Median: $442 [IQR 0-993] Complex continuing care: Mean: $1464 (SD 12,026) Long-term care: Mean: $22 (SD 476) Rehabilitation: Mean: $4740 (SD 15,174) Homecare: Mean: $1186 (SD 3177), Median: $0 [IQR 0-712] Outpatient clinics: Mean $3822 (SD 4057), Median: $2435 [IQR 767-6140] Laboratory (OHIP): Mean $227 (SD 372), Median: $0 [IQR 0-329] Drugs (Ontario Drug Benefit Program): Mean $4537 (SD 9606), Median: $0 [IQR 0-4148] Physician billings: Mean $26,651 (SD 18,306), Median: $23,283 [IQR 13,288-35,313] |
| Hess  (2021) | Post-cardiotomy MCS | NR | NR | - | - | NR |
| Huesch (2018) | Physician procedural code for ECMO | NR | NR | - | - | NR |
| Jaamaa-Holmberg (2020) | VA ECMO due to CS or refractory CA | *ECPR patients:* Median [IQR] $31,006 ($17,143) (3.5% discount rate | NR | - | - | In-hospital costs *(per-patient)*: Median: $187,859 [IQR 217-306] 1y hospital related costs *(per-patient)*: Median: $23,857 [IQR 41-524] |
| Kim  (2020) | CA patients with a CPR procedure claim code | NR | HP | - | - | Short term hospital cost: Mean: $24,449 (SD 18,771), Median: $19,434 [IQR 11,381-319,795] Long term hospital cost: Mean: $39,081 (SD 40,771), Median: $26,128 [IQR 10,701-54,637] |
| Mayer (2022) | ECMO > 72 hours | NR | NR | - | - | NR |
| Nuqali (2022) | CS | NR | NR | - | - | NR |
| Oh  (2022) | ECMO survivors alive 365 days after ECMO initiation | NR | H; HP; Other (self-payment) | - | Total Healthcare Cost for 1-year *(all ECMO survivors)*: Median: $58,791 [IQR 32,662 - 110,356]  Self-Payment by Patient for 1-year *(all ECMO survivors)*: Median: $5,028 [IQR 2,382 - 10,286]  NHIS Insurance Coverage for 1-year *(all ECMO survivors)*: Median: $52,804 [IQR 29,244 - 98,785] | Total healthcare cost for one year: Median: $58,791 [IQR 32,662-110,356]  Self-payment by patient for 1y: Median: $5027 [IQR 2382-10,286]  NHIS insurance coverage for 1y: Median: $52805 [IQR 29,244-98,785] |
| Oude Lansink-Hartgring (2023) | ECMO (excluding bridging procedure or during surgery) | *All ECMO patients:* $26,414 (4.0% discount rate) | H; S | - | Total costs at 1y *(all ECMO patients)*: Mean: $243,747 (SD 252,181) (95% CI, 209,224 - 243,820) | Hospital costs: Mean $170,961 (SD 154,341) Follow up costs: Mean $64064 (SD 77,530) [95% CI, 50,254-76,089] Costs of absenteeism: Mean $8721 (SD 20304) [95% CI, 5492-12047] |
| Peek (2010) | Severe, but potentially reversible, respiratory failure or uncompensated hypercapnia | *Patients allocated to ECMO:* $42,305 (95% CI, $16,749-$130,088) (3.5% discount rate) | H; HP; S | - | Total cost at 6m (*patients allocated to ECMO)*: Mean: $162,564 | Cost per CESAR trial participant (N=90) *Discharge to 6 months:* Travel home after discharge: Mean: $40 GP Surgery services: Mean: $130 Phone calls to NHS professionals: Mean $9 Visits to nurse: Mean: $187 Visits to physiotherapist: Mean $259 Visits to occupational therapist: Mean: $55 Counselling services: Mean: $18 Other nursing, therapy and social services: Mean: $171 Inpatient stay: Mean: $923 Outpatient visits: Mean: $275 Other hospital services: Mean: $ 20 Nursing home and residential care: Mean: $44 Medication: Mean: $261 Aids and adaptations: Mean: $42 Hospital transport (discharge to follow up): Mean: $12 Unpaid help from family/friends: Mean: $9519 |
| Sanaiha (2019) | ECLS | NR | H; HP | 90d readmission costs (all ECLS patients): Mean: $40,881 (SD $2,828) | - | Index costs: Mean: $251,200 (SD 237,059) |
| Scotti (2015) | CS | NR | HP | 30d: Mean $62150 90d: Mean $43445 | - | NR |
| Tashtish (2020) | CS | NR | H; HP | 30d: Hospitalization charges for readmissions: Median $92316 | Cost of hospitalization *(overall group)*: Mean $1,355,411  Cost of hospitalization *(overall group)*: Median: $925,193 [IQR 492,516-1,736,070]  Cost of hospitalization *(readmitted group)*: Mean: $1,547,647   Cost of hospitalization *(readmitted group)*: Median: $1,181,685 [IQR 632,289-2,063,215] | NR |
| Varvoutis (2023) | ECMO | NR | H | - | - | Total inpatient charges: Mean: $1,021,168 (SD 526,912) Total inpatient costs: Mean: $261,013 (SD 120,852) Total inpatient charges (30d outcomes): Mean: $1,0443,43 (SD 517,438) Total inpatient costs (30d outcomes): Mean: $268,294 (SD 119,475) Total inpatient charges (60d outcomes): Mean: $1,062,900 (SD 509,145) Total inpatient costs (60d outcomes): Mean: $273,491 (SD 117,133) |
| Vetrovec (2021) | Patients with acute MI (AMICS) | NR | HP | NR | Total episode costs (index facility and post-index costs): Mean *(ECMO cohort)* $214,331 | *Index and post-index 45-day average costs:* SNF costs: Mean: $8687 Index facility costs: Mean: $191,089 Inpatient costs: Mean: $111,985 Outpatient costs: Mean: $1755 ER costs: Mean: $2183 Home health costs: Mean: $3835 Post-index costs: Mean: $71,739 |

*AHP,* Allied Health Professional*; CA,* cardiac arrest*; CS,* cardiogenic shock*; ECLS,* extracorporeal life support*; ER,* Emergency room*; ES,* electrical storm*; H,* Hospital*; HP,* Healthcare Payer*; MCS,* mechanical circulatory support*; MI,* myocardial infarction*; NR,* not reported*; OHCA,* out-of-hospital cardiac arrest*; QALY*, quality-adjusted life-year*; S,* Societal*; SNF,* skilled nursing facility*; VA,* venoarterial ECMO*; VV,* venovenous ECMO*.*

# Supplemental Table S5. Summary Table of Pooled Proportions

| **Outcome** | **N studies** | **Proportion (95% CI)** | **Tau^2^** | **I [95% CI]** | **Q** | **Prediction interval** |
| --- | --- | --- | --- | --- | --- | --- |
| In-hospital mortality | 18 | 0.47 [0.42-0.53] | 0.2 | 97.7% [97.1%-98.2%] | 738.23, p=0.00000 | [0.24-0.71] |
| 30-day mortality | 3 | 0.57 [0.36-0.77] | 0.5 | 99.4% [99.2%-99.6%] | 361.75, p=0.00000 | [0.00-1.00] |
| 6-month mortality | 2 | 0.68 [0.42-0.86] | 0.6 | 97.4% [93.6%-99.0%] | 39.11, p=0.00000 | [0.26-0.93] |
| 1-year mortality | 9 | 0.62 [0.51-0.72[ | 0.4 | 98.7% [98.4%-99.0%] | 639.09, p=0.00000 | [0.24-0.89] |
| 30-day readmission | 8 | 0.20 [0.14-0.28] | 0.4 | 99.7% [99.6%-99.7%] | 2096.15, p=0.00000 | [0.05-0.55] |
| 1-year readmission | 5 | 0.16 [0.03-0.51] | 3.7 | 99.5% [99.4%-99.6%] | 870.28, p=0.00000 | [0.00-0.99] |

# Supplemental Table S6. Quality Assessment of Included Studies

|  | **Nuqali  (2022)** | **Scotti (2015)** | **Huesch (2018)** | **Sanaiha  (2019)** | **Tashtish  (2020)** | **Christian-Miller**  **(2020)** | **Vetrovec (2021)** | **Hess  (2021)** |
| --- | --- | --- | --- | --- | --- | --- | --- | --- |
| Title | Yes | Yes | Yes | Yes | Yes | Yes | Yes | Yes |
| Abstract | Yes | Yes | Yes | Yes | Yes | Yes | Yes | Yes |
| Introduction - Background | Yes | Yes | Yes | Yes | Yes | Yes | Yes | Yes |
| Introduction - Study objectives | Yes | Yes | Yes | Yes | Yes | Yes | Yes | Yes |
| Methods - Study population | Yes | Yes | Yes | Yes | Yes | Yes | Yes | Yes |
| Methods - Study population  *Appropriateness* | Appropriate | Appropriate | Appropriate | Appropriate | Appropriate | Appropriate | Appropriate | Appropriate |
| Methods - Setting and location | Yes | Yes | Yes | Yes | Yes | Yes | Yes | Yes |
| Methods - Setting and location *Appropriateness* | Appropriate | Appropriate | Appropriate | Appropriate | Appropriate | Appropriate | Appropriate | Appropriate |
| Methods - Comparators | Not applicable | Yes | Not applicable | Not applicable | Not applicable | Not applicable | Yes | Yes |
| Methods – Comparators *Appropriateness* | Not applicable | Appropriate | Not applicable | Not applicable | Not applicable | Not applicable | Appropriate | Appropriate |
| Methods - Perspective | Not applicable | Yes | Not applicable | Yes | Yes | Yes | Yes | Not applicable |
| Methods – Perspective  *Appropriateness* | Not applicable | Appropriate | Not applicable | Appropriate | Appropriate | Appropriate | Appropriate | Not applicable |
| Methods - Time horizon | Not applicable | Yes | Not applicable | Yes | Yes | No | Yes | Not applicable |
| Methods - Time horizon  *Appropriateness* | Not applicable | Appropriate | Not applicable | Appropriate | Appropriate | Not applicable | Appropriate | Not applicable |
| Methods - Common patient clinical pathway | Yes | Yes | Yes | Yes | Yes | Yes | Yes | Yes |
| Methods - Common patient clinical pathway *Appropriateness* | Appropriate | Appropriate | Appropriate | Appropriate | Appropriate | Appropriate | Appropriate | Appropriate |
| Methods - Costing methodology | Not applicable | Yes | Not applicable | No | No | No | Yes | Not applicable |
| Methods - Costing methodology *Appropriateness* | Not applicable | Appropriate | Not applicable | Not applicable | Not applicable | Not applicable | Appropriate | Not applicable |
| Methods - Currency, price date, and conversion | Not applicable | Yes | Not applicable | Yes | Yes | No | Yes | Not applicable |
| Methods - Currency, price date, and conversion *Appropriateness* | Not applicable | Appropriate | Not applicable | Appropriate | Appropriate | Not applicable | Appropriate | Not applicable |
| Resource identification - Identification of resource elements | Not applicable | No | Not applicable | Yes | No | No | Yes | Not applicable |
| Resource identification - Identification of resource elements *Appropriateness* | Not applicable | Not applicable | Not applicable | Appropriate | Not applicable | Not applicable | Appropriate | Not applicable |
| Resource identification - Methods/ tools used to identify resource items | Yes | Yes | Yes | Yes | Yes | Yes | Yes | Yes |
| Resource identification - Methods/ tools used to identify resource items *Appropriateness* | Appropriate | Appropriate | Appropriate | Appropriate | Appropriate | Appropriate | Appropriate | Appropriate |
| Resource identification - Sample size calculation | Not applicable | Not applicable | Not applicable | Not applicable | Not applicable | Not applicable | Yes | Not applicable |
| Resource identification - Sample size calculation *Appropriateness* | Not applicable | Not applicable | Not applicable | Not applicable | Not applicable | Not applicable | Appropriate | Not applicable |
| Resource identification - Resource items are clearly identified and listed | Not applicable | No | Not applicable | No | No | No | Yes | Not applicable |
| Resource identification - Resource items are clearly identified and listed *Appropriateness* | Not applicable | Not applicable | Not applicable | Not applicable | Not applicable | Not applicable | Appropriate | Not applicable |
| Resource identification - Classification of cost items | Not applicable | No | Not applicable | No | Yes | No | Yes | Not applicable |
| Resource identification - Classification of cost items *Appropriateness* | Not applicable | Not applicable | Not applicable | Not applicable | Appropriate | Not applicable | Appropriate | Not applicable |
| Resource identification - Identification of joint costs | Not applicable | No | Not applicable | Yes | No | Yes | Yes | Not applicable |
| Resource identification - Identification of joint costs *Appropriateness* | Not applicable | Not applicable | Not applicable | Appropriate | Not applicable | Appropriate | Appropriate | Not applicable |
| Resource identification - Subgroup analysis | Yes | Yes | Yes | Yes | Yes | Yes | Yes | Yes |
| Resource identification - Subgroup analysis *Appropriateness* | Appropriate | Appropriate | Appropriate | Appropriate | Appropriate | Appropriate | Appropriate | Appropriate |
| Resource identification - Assumption regarding resource use measurement | Not applicable | Not applicable | Not applicable | Not applicable | No | Not applicable | Yes | Not applicable |
| Resource identification - Assumption regarding resource use measurement *Appropriateness* | Not applicable | Not applicable | Not applicable | Not applicable | Not applicable | Not applicable | Appropriate | Not applicable |
| Resource identification - Dealing with uncertainties | Yes | Not applicable | Not applicable | Yes | No | Not applicable | Yes | Not applicable |
| Resource identification - Dealing with uncertainties *Appropriateness* | Appropriate | Not applicable | Not applicable | Appropriate | Not applicable | Not applicable | Appropriate | Not applicable |
| Resource valuation - Valuing resource items | Not applicable | Yes | Not applicable | Yes | No | No | No | Not applicable |
| Resource valuation - Valuing resource items *Appropriateness* | Not applicable | Appropriate | Not applicable | Appropriate | Not applicable | Not applicable | Not applicable | Not applicable |
| Resource valuation - Direct measurement | Not applicable | Yes | Not applicable | No | No | No | No | Not applicable |
| Resource valuation - Direct measurement *Appropriateness* | Not applicable | Appropriate | Not applicable | Not applicable | Not applicable | Not applicable | Not applicable | Not applicable |
| Resource valuation - Prices for marketable items | Not applicable | No | Not applicable | Not applicable | No | Not applicable | No | Not applicable |
| Resource valuation - Prices for marketable items *Appropriateness* | Not applicable | Not applicable | Not applicable | Not applicable | Not applicable | Not applicable | Not applicable | Not applicable |
| Resource valuation - Fees/tariffs/charges | Not applicable | No | Not applicable | Not applicable | No | Not applicable | No | Not applicable |
| Resource valuation - Fees/tariffs/charges *Appropriateness* | Not applicable | Not applicable | Not applicable | Not applicable | Not applicable | Not applicable | Not applicable | Not applicable |
| Resource valuation - Sources of cost estimates | Not applicable | Yes | Not applicable | Yes | Yes | Yes | Yes | Not applicable |
| Resource valuation - Sources of cost estimates *Appropriateness* | Not applicable | Appropriate | Not applicable | Appropriate | Appropriate | Appropriate | Appropriate | Not applicable |
| Resource valuation - Assumptions regarding attaching monetary value to resource use | Not applicable | Not applicable | Not applicable | Yes | No | Not applicable | Not applicable | Not applicable |
| Resource valuation - Assumptions regarding attaching monetary value to resource use *Appropriateness* | Not applicable | Not applicable | Not applicable | Appropriate | Not applicable | Not applicable | Not applicable | Not applicable |
| Results - Study parameters | Yes | Yes | Yes | Yes | Yes | Yes | Yes | Yes |
| Results - Summary of main results | Yes | Yes | Yes | Yes | Yes | Yes | Yes | Yes |
| Results - Effects of uncertainty | No | No | No | No | No | No | No | No |
| Results - Effect of health system-specific factors | No | Yes | No | No | Yes | No | No | No |
| Discussion - Study findings, limitations, generalizability, and current knowledge. | Yes | Yes | Yes | Yes | Yes | Yes | Yes | Yes |
| Other relevant information - Source of funding | Yes | Yes | No | No | Yes | No | Yes | No |
| Other relevant information - Conflicts of interest | Yes | Yes | Yes | Yes | Yes | Yes | Yes | Yes |

|  | **Mayer  (2022)** | **Briasoulis (2023)** | **Varvoutis (2023)** | **Banning  (2023)** | **Peek  (2010)** | **Oude Lansink-Hartgring (2023)** | **Chen  (2017)** | **Kim (2020)** |
| --- | --- | --- | --- | --- | --- | --- | --- | --- |
| Title | Yes | Yes | Yes | Yes | Yes | Yes | Yes | Yes |
| Abstract | Yes | Yes | Yes | Yes | Yes | Yes | Yes | Yes |
| Introduction - Background | Yes | Yes | Yes | Yes | Yes | Yes | Yes | Yes |
| Introduction - Study objectives | Yes | Yes | Yes | Yes | Yes | Yes | Yes | Yes |
| Methods - Study population | Yes | Yes | Yes | Yes | Yes | Yes | Yes | Yes |
| Methods - Study population  Appropriateness | Appropriate | Appropriate | Appropriate | Appropriate | Appropriate | Appropriate | Appropriate | Appropriate |
| Methods - Setting and location | Yes | Yes | Yes | Yes | Yes | Yes | Yes | Yes |
| Methods - Setting and location Appropriateness | Appropriate | Appropriate | Appropriate | Appropriate | Appropriate | Appropriate | Appropriate | Appropriate |
| Methods - Comparators | Not applicable | Yes | Not applicable | Yes | Yes | Not applicable | Yes | Yes |
| Methods – Comparators Appropriateness | Not applicable | Appropriate | Not applicable | Appropriate | Appropriate | Not applicable | Appropriate | Appropriate |
| Methods - Perspective | Not applicable | Yes | Yes | Not applicable | Yes | Yes | Yes | Yes |
| Methods – Perspective  Appropriateness | Not applicable | Appropriate | Appropriate | Not applicable | Appropriate | Appropriate | Appropriate | Appropriate |
| Methods - Time horizon | Yes | Yes | Yes | Not applicable | Yes | Yes | Yes | Yes |
| Methods - Time horizon  Appropriateness | Appropriate | Appropriate | Appropriate | Not applicable | Appropriate | Appropriate | Appropriate | Appropriate |
| Methods - Common patient clinical pathway | Yes | Yes | Yes | Yes | Yes | Yes | Yes | Yes |
| Methods - Common patient clinical pathway Appropriateness | Appropriate | Appropriate | Appropriate | Appropriate | Appropriate | Appropriate | Appropriate | Appropriate |
| Methods - Costing methodology | Not applicable | No | Yes | Not applicable | Yes | Yes | No | Yes |
| Methods - Costing methodology Appropriateness | Not applicable | Not applicable | Appropriate | Not applicable | Appropriate | Appropriate | Not applicable | Appropriate |
| Methods - Currency, price date, and conversion | Not applicable | No | Yes | Not applicable | Yes | Yes | Yes | Yes |
| Methods - Currency, price date, and conversion Appropriateness | Not applicable | Not applicable | Appropriate | Not applicable | Appropriate | Appropriate | Appropriate | Appropriate |
| Resource identification - Identification of resource elements | Not applicable | No | No | Not applicable | Yes | Yes | Yes | No |
| Resource identification - Identification of resource elements Appropriateness | Not applicable | Not applicable | Not applicable | Not applicable | Appropriate | Appropriate | Appropriate | Not applicable |
| Resource identification - Methods/ tools used to identify resource items | Yes | Yes | Yes | Yes | Yes | Yes | Yes | Yes |
| Resource identification - Methods/ tools used to identify resource items Appropriateness | Appropriate | Appropriate | Appropriate | Appropriate | Appropriate | Appropriate | Appropriate | Appropriate |
| Resource identification - Sample size calculation | Not applicable | Not applicable | Not applicable | Yes | Yes | Not applicable | Yes | Yes |
| Resource identification - Sample size calculation Appropriateness | Not applicable | Not applicable | Not applicable | Appropriate | Appropriate | Not applicable | Appropriate | Appropriate |
| Resource identification - Resource items are clearly identified and listed | Not applicable | No | No | Not applicable | Yes | Yes | No | No |
| Resource identification - Resource items are clearly identified and listed Appropriateness | Not applicable | Not applicable | Not applicable | Not applicable | Appropriate | Appropriate | Not applicable | Not applicable |
| Resource identification - Classification of cost items | Not applicable | No | Yes | Not applicable | Yes | Yes | No | No |
| Resource identification - Classification of cost items Appropriateness | Not applicable | Not applicable | Appropriate | Not applicable | Appropriate | Appropriate | Not applicable | Not applicable |
| Resource identification - Identification of joint costs | Not applicable | No | Yes | Not applicable | Yes | Yes | No | Not applicable |
| Resource identification - Identification of joint costs Appropriateness | Not applicable | Not applicable | Appropriate | Not applicable | Appropriate | Appropriate | Not applicable | Not applicable |
| Resource identification - Subgroup analysis | Yes | Yes | Yes | Yes | Yes | Yes | Yes | Yes |
| Resource identification - Subgroup analysis Appropriateness | Appropriate | Appropriate | Appropriate | Appropriate | Appropriate | Appropriate | Appropriate | Appropriate |
| Resource identification - Assumption regarding resource use measurement | Not applicable | Not applicable | Yes | Not applicable | Yes | Yes | Yes | Not applicable |
| Resource identification - Assumption regarding resource use measurement Appropriateness | Not applicable | Not applicable | Appropriate | Not applicable | Appropriate | Appropriate | Appropriate | Not applicable |
| Resource identification - Dealing with uncertainties | Not applicable | Not applicable | Yes | Not applicable | Yes | Yes | Yes | Not applicable |
| Resource identification - Dealing with uncertainties Appropriateness | Not applicable | Not applicable | Appropriate | Not applicable | Appropriate | Appropriate | Appropriate | Not applicable |
| Resource valuation - Valuing resource items | Not applicable | Not applicable | Yes | Not applicable | Yes | Yes | No | No |
| Resource valuation - Valuing resource items Appropriateness | Not applicable | Not applicable | Appropriate | Not applicable | Appropriate | Appropriate | Not applicable | Not applicable |
| Resource valuation - Direct measurement | Not applicable | No | Yes | Not applicable | Yes | Yes | No | No |
| Resource valuation - Direct measurement Appropriateness | Not applicable | Not applicable | Appropriate | Not applicable | Appropriate | Appropriate | Not applicable | Not applicable |
| Resource valuation - Prices for marketable items | Not applicable | Not applicable | No | Not applicable | No | Not applicable | No | Not applicable |
| Resource valuation - Prices for marketable items Appropriateness | Not applicable | Not applicable | Not applicable | Not applicable | Not applicable | Not applicable | Not applicable | Not applicable |
| Resource valuation - Fees/tariffs/charges | Not applicable | Not applicable | Yes | Not applicable | Yes | Not applicable | Not applicable | Not applicable |
| Resource valuation - Fees/tariffs/charges Appropriateness | Not applicable | Not applicable | Appropriate | Not applicable | Appropriate | Not applicable | Not applicable | Not applicable |
| Resource valuation - Sources of cost estimates | Not applicable | Yes | Yes | Not applicable | Yes | Yes | Yes | Yes |
| Resource valuation - Sources of cost estimates Appropriateness | Not applicable | Appropriate | Appropriate | Not applicable | Appropriate | Appropriate | Appropriate | Appropriate |
| Resource valuation - Assumptions regarding attaching monetary value to resource use | Not applicable | Not applicable | No | Not applicable | Yes | Yes | Not applicable | Not applicable |
| Resource valuation - Assumptions regarding attaching monetary value to resource use Appropriateness | Not applicable | Not applicable | Not applicable | Not applicable | Appropriate | Appropriate | Not applicable | Not applicable |
| Results - Study parameters | Yes | Yes | Yes | Yes | Yes | Yes | Yes | Yes |
| Results - Summary of main results | Yes | Yes | Yes | Yes | Yes | Yes | Yes | Yes |
| Results - Effects of uncertainty | No | No | No | No | Yes | Yes | No | No |
| Results - Effect of health system-specific factors | No | No | Yes | No | Yes | Yes | Yes | Yes |
| Discussion - Study findings, limitations, generalizability, and current knowledge. | Yes | Yes | Yes | Yes | Yes | Yes | Yes | Yes |
| Other relevant information - Source of funding | Yes | Yes | Yes | Yes | Yes | Yes | No | Yes |
| Other relevant information - Conflicts of interest | Yes | Yes | Yes | Yes | Yes | Yes | Yes | Yes |

|  | **Oh (2022)** | **Jaamaa-Holmberg (2020)** | **Fernando (2019)** | **Bak (2024)** | **Desch (2024)** | **Chan (2024)** | **Duraes-Campos (2024)** | **Delnoij (2024)** |
| --- | --- | --- | --- | --- | --- | --- | --- | --- |
| Title | Yes | Yes | Yes | Yes | Yes | Yes | Yes | Yes |
| Abstract | Yes | Yes | Yes | Yes | No | Yes | Yes | Yes |
| Introduction - Background | Yes | Yes | Yes | Yes | Yes | Yes | Yes | Yes |
| Introduction - Study objectives | Yes | Yes | Yes | Yes | Yes | Yes | Yes | Yes |
| Methods - Study population | Yes | Yes | Yes | Yes | Yes | Yes | Yes | Yes |
| Methods - Study population  *Appropriateness* | Appropriate | Appropriate | Appropriate | Appropriate | Appropriate | Appropriate | Appropriate | Appropriate |
| Methods - Setting and location | Yes | Yes | Yes | Yes | No | Yes | Yes | Yes |
| Methods - Setting and location *Appropriateness* | Appropriate | Appropriate | Appropriate | Not appropriate | Not appropriate | Appropriate | Not appropriate | Not appropriate |
| Methods - Comparators | Not applicable | Not applicable | Not applicable | Not applicable | Yes | Not applicable | Not applicable | Yes |
| Methods – Comparators *Appropriateness* | Not applicable | Not applicable | Not applicable | Not applicable | Appropriate | Not applicable | Not applicable | Appropriate |
| Methods - Perspective | Yes | No | Yes | Not Applicable | Not applicable | Not applicable | Not applicable | Yes |
| Methods – Perspective  *Appropriateness* | Appropriate | Not applicable | Appropriate | Not applicable | Not applicable | Not applicable | Not applicable | Appropriate |
| Methods - Time horizon | Yes | Yes | Yes | Not Applicable | Not applicable | Not applicable | Not applicable | Yes |
| Methods - Time horizon  *Appropriateness* | Appropriate | Appropriate | Appropriate | Not applicable | Not applicable | Not applicable | Not applicable | Appropriate |
| Methods - Common patient clinical pathway | Yes | Yes | Yes | Yes | Yes | Yes | Yes | Yes |
| Methods - Common patient clinical pathway *Appropriateness* | Appropriate | Appropriate | Appropriate | Appropriate | Appropriate | Appropriate | Appropriate | Appropriate |
| Methods - Costing methodology | Yes | Yes | Yes | Not applicable | Not applicable | Not applicable | Not applicable | Yes |
| Methods - Costing methodology *Appropriateness* | Appropriate | Appropriate | Appropriate | Not applicable | Not applicable | Not applicable | Not applicable | Appropriate |
| Methods - Currency, price date, and conversion | Yes | Yes | Yes | Not applicable | Not applicable | Not applicable | Not applicable | Yes |
| Methods - Currency, price date, and conversion *Appropriateness* | Appropriate | Appropriate | Appropriate | Not applicable | Not applicable | Not applicable | Not applicable | Appropriate |
| Resource identification - Identification of resource elements | Yes | Yes | Yes | Not applicable | Yes | Not applicable | Not applicable | Yes |
| Resource identification - Identification of resource elements *Appropriateness* | Appropriate | Appropriate | Appropriate | Not applicable | Appropriate | Not applicable | Not applicable | Appropriate |
| Resource identification - Methods/ tools used to identify resource items | Yes | Yes | Yes | Yes | No | Not applicable | Not applicable | Yes |
| Resource identification - Methods/ tools used to identify resource items *Appropriateness* | Appropriate | Appropriate | Appropriate | Appropriate | Not applicable | Not applicable | Not applicable | Appropriate |
| Resource identification - Sample size calculation | Not applicable | Yes | Not applicable | Yes | Yes | Not applicable | Not applicable | Yes |
| Resource identification - Sample size calculation *Appropriateness* | Not applicable | Appropriate | Not applicable | Appropriate | Appropriate | Not applicable | Not applicable | Appropriate |
| Resource identification - Resource items are clearly identified and listed | No | No | Yes | Not applicable | Not applicable | Not applicable | Not applicable | Yes |
| Resource identification - Resource items are clearly identified and listed *Appropriateness* | Not applicable | Not applicable | Appropriate | Not applicable | Not applicable | Not applicable | Not applicable | Appropriate |
| Resource identification - Classification of cost items | Yes | No | Yes | Not applicable | Not applicable | Not applicable | Not applicable | Yes |
| Resource identification - Classification of cost items *Appropriateness* | Appropriate | Not applicable | Appropriate | Not applicable | Not applicable | Not applicable | Not applicable | Appropriate |
| Resource identification - Identification of joint costs | Yes | Yes | Yes | Not applicable | Not applicable | Not applicable | Not applicable | Not applicable |
| Resource identification - Identification of joint costs *Appropriateness* | Appropriate | Appropriate | Appropriate | Not applicable | Not applicable | Not applicable | Not applicable | Not applicable |
| Resource identification - Subgroup analysis | Yes | Yes | Yes | Yes | Yes | Yes | Yes | Not applicable |
| Resource identification - Subgroup analysis *Appropriateness* | Appropriate | Appropriate | Appropriate | Appropriate | Appropriate | Appropriate | Appropriate | Not applicable |
| Resource identification - Assumption regarding resource use measurement | Not applicable | Not applicable | Not applicable | Not applicable | Not applicable | Not applicable | Not applicable | Not applicable |
| Resource identification - Assumption regarding resource use measurement *Appropriateness* | Not applicable | Not applicable | Not applicable | Not applicable | Not applicable | Not applicable | Not applicable | Not applicable |
| Resource identification - Dealing with uncertainties | Yes | Not applicable | No | Not applicable | Not applicable | Yes | Not applicable | Yes |
| Resource identification - Dealing with uncertainties *Appropriateness* | Appropriate | Not applicable | Not applicable | Not applicable | Not applicable | Appropriate | Not applicable | Appropriate |
| Resource valuation - Valuing resource items | Yes | No | Yes | Not applicable | Not applicable | Not applicable | Not applicable | Yes |
| Resource valuation - Valuing resource items *Appropriateness* | Appropriate | Not applicable | Appropriate | Not applicable | Not applicable | Not applicable | Not applicable | Appropriate |
| Resource valuation - Direct measurement | No | No | No | Not applicable | Not applicable | Not applicable | Not applicable | Yes |
| Resource valuation - Direct measurement *Appropriateness* | Not applicable | Not applicable | Not applicable | Not applicable | Not applicable | Not applicable | Not applicable | Appropriate |
| Resource valuation - Prices for marketable items | Not applicable | Not applicable | No | Not applicable | Not applicable | Not applicable | Not applicable | Not applicable |
| Resource valuation - Prices for marketable items *Appropriateness* | Not applicable | Not applicable | Not applicable | Not applicable | Not applicable | Not applicable | Not applicable | Not applicable |
| Resource valuation - Fees/tariffs/charges | Not applicable | Not applicable | No | Not applicable | Not applicable | Not applicable | Not applicable | Not applicable |
| Resource valuation - Fees/tariffs/charges *Appropriateness* | Not applicable | Not applicable | Not applicable | Not applicable | Not applicable | Not applicable | Not applicable | Not applicable |
| Resource valuation - Sources of cost estimates | Yes | Yes | Yes | Not applicable | Not applicable | Not applicable | Not applicable | Yes |
| Resource valuation - Sources of cost estimates *Appropriateness* | Appropriate | Appropriate | Appropriate | Not applicable | Not applicable | Not applicable | Not applicable | Appropriate |
| Resource valuation - Assumptions regarding attaching monetary value to resource use | Not applicable | Not applicable | No | Not applicable | Not applicable | Not applicable | Not applicable | Yes |
| Resource valuation - Assumptions regarding attaching monetary value to resource use *Appropriateness* | Not applicable | Not applicable | Not applicable | Not applicable | Not applicable | Not applicable | Not applicable | Appropriate |
| Results - Study parameters | Yes | Yes | Yes | Yes | Yes | Yes | No | No |
| Results - Summary of main results | Yes | Yes | Yes | Yes | Yes | Yes | Yes | Yes |
| Results - Effects of uncertainty | Yes | Yes | No | Yes | Yes | Yes | No | Yes |
| Results - Effect of health system-specific factors | Yes | Yes | Yes | Yes | Yes | Yes | Yes | Yes |
| Discussion - Study findings, limitations, generalizability, and current knowledge. | Yes | Yes | Yes | Yes | Yes | Yes | Yes | Yes |
| Other relevant information - Source of funding | Yes | Yes | Yes | Yes | Yes | Yes | Yes | Yes |
| Other relevant information - Conflicts of interest | Yes | Yes | Yes | Yes | Yes | Yes | Yes | Yes |

# Supplemental Appendix S1 - Pre-defined Data Extraction Form

| **General Information** | |
| --- | --- |
| 1. Study ID (#) |  |
| 1. Author(s) |  |
| 1. Year of Publication |  |
| 1. Title |  |
| 1. Country where the study was conducted | - Multinational - United States of America (USA) - United Kingdom (UK) - Australia - New Zealand - Taiwan - Scotland - Korea - Other |
| **Characteristics of included studies** | |
| *Methods* | |
| 1. Study Design (select all that apply) | - Prospective Cohort Study - Retrospective Cohort Study - Randomised Controlled Trial (RCT) - Non-randomised experimental study - Case-Control Study - Cross-Sectional Study - Economic evaluation - Other |
| 1. Study Setting (check all that apply) | - Metro - Regional - Rural - Not reported |
| 1. Site | - Multi-centre (≥2) - Single centre - Not reported |
| 1. If multi-centre, how many centres? |  |
| 1. Study Population |  |
| 1. Exclusion Criteria |  |
| 1. Comparator |  |
| 1. Type of ECMO | - Venoarterial (VA) - Venovenous (VV) - Extracorporeal Cardiopulmonary Resuscitation (ECPR) - Not reported - Other |
| 1. What was the indication for ECMO use? | - Cardiac - Respiratory - Cardiopulmonary resuscitation or cardiac arrest - Not reported - Other |
| 1. Funding source | - Industry (e.g. pharma, medical device company) - Not-for-profit (e.g. philanthropic, charity) - Government (e.g. NHMRC, MRFF) - Other |

1. **Duration of ECMO support (total)**

|  | **Mean (SD)** | **Median [IQR]** |
| --- | --- | --- |
| ECMO (days) |  |  |
| Comparator (days) |  |  |

1. **Intervention and Comparator**

|  | **ECMO** | **Comparator** |
| --- | --- | --- |
| Number of pts |  |  |
| In-hospital mortality (n/N) |  |  |
| In-hospital mortality (%) |  |  |

1. **Mortality Rate**

|  | **Time point** | **ECMO (n/N)** | **ECMO (%)** | **Comparator (n/N)** | **Comparator (%)** |
| --- | --- | --- | --- | --- | --- |
| **1** |  |  |  |  |  |
| **2** |  |  |  |  |  |
| **3** |  |  |  |  |  |

1. **Lost to follow up rate**

|  | **Time point** | **ECMO (n/N)** | **ECMO (%)** | **Comparator (n/N)** | **Comparator (%)** |
| --- | --- | --- | --- | --- | --- |
| **1** |  |  |  |  |  |
| **2** |  |  |  |  |  |
| **3** |  |  |  |  |  |

1. **Number of readmissions**

|  | **Type of readmission (e.g. ED, acute care, rehab)** | **Time point** | **ECMO (n/N)** | **ECMO (%)** | **ECMO duration (mean (SD), median [IQR])** | **Comparator (n/N)** | **Comparator (%)** | **Comparator duration (mean (SD), median [IQR])** |
| --- | --- | --- | --- | --- | --- | --- | --- | --- |
| **1** |  |  |  |  |  |  |  |  |
| **2** |  |  |  |  |  |  |  |  |
| **3** |  |  |  |  |  |  |  |  |

1. **Mean number of readmissions**

|  | **ECMO: Mean [95% CI]** | **Comparator: Mean [95% CI]** |
| --- | --- | --- |
| **1** |  |  |
| **2** |  |  |
| **3** |  |  |

1. **Resource use**

|  | **Type of resource** | **Time point** | **ECMO (mean visits/events)** | **Comparator (mean visits/events)** |
| --- | --- | --- | --- | --- |
| **1** |  |  |  |  |
| **2** |  |  |  |  |
| **3** |  |  |  |  |

| **Results** | |
| --- | --- |
| 1. Reasons for readmission |  |
| 1. Follow-up time point:   (related to health resource use) | - 30 days - 3 months - 6 months - 1 year - 2 years - Other |
| 1. Sensitivity analysis   Indicate whether any sensitivity analyses were conducted | - Yes - No |
| *Costs* | |
| 1. Perspective (i.e. who incurs the cost)   Societal: All the costs, pt travelling to hospital/missing work | - Hospital - Healthcare payer - Societal - Other |
| 1. Currency (including year): |  |
| 1. Year of collected cost: |  |
| 1. Year of reported cost: |  |

1. **Total Cost of Treatment**

Enter the type of cost (e.g. index admission costs, readmission cost) followed by the mean (SD) or median [IQR] in the corresponding rows.

|  | **Type of cost (e.g. index admission, rehab, total costs)** | **ECMO: Mean (SD) or mean [95% CI]** | **ECMO: Median [IQR]** | **Comparator: Mean (SD) or mean [95% CI]** | **Comparator: Median [IQR]** |
| --- | --- | --- | --- | --- | --- |
| **1** |  |  |  |  |  |
| **2** |  |  |  |  |  |
| **3** |  |  |  |  |  |
| **4** |  |  |  |  |  |
| **5** |  |  |  |  |  |

# Supplemental Appendix S2 - Quality Assessment Template

| **Items** | **Guidance for Reporting** | **Yes** | **No** | **Appropriate** | **Not appropriate** |
| --- | --- | --- | --- | --- | --- |
| **TITLE** | | | | | |
| Title | Identify the study as a costing study and specify the population/setting. |  |  |  |  |
| **ABSTRACT** | | | | | |
| Abstract | Provide a structured summary that highlights context, key methods, results and alternative analyses. |  |  |  |  |
| **INTRODUCTION** | | | | | |
| Background | Give the context for the study, the study question and its practical relevance for decision making in policy or practice. |  |  |  |  |
| Study objectives | The purpose and the objectives of the study are explicit and clear. |  |  |  |  |
| **METHODS** | |  |  |  |  |
| Study population | Describe characteristics of the study population (such as age range, demographics, socioeconomic, or clinical characteristics). |  |  |  |  |
| Setting and location | Provide relevant contextual information that may influence findings, including institutional characteristics (e.g., rural/urban location, capacity, and occupancy rates) |  |  |  |  |
| Comparators | Describe the interventions or strategies being compared and why chosen. |  |  |  |  |
| Perspective | State the perspective(s) adopted by the study and why chosen. |  |  |  |  |
| Time horizon | State the time horizon for the study and why appropriate. |  |  |  |  |
| Common patient clinical pathway | Treatment thresholds, cut-off points, indication for hospitalization, operation, etc. |  |  |  |  |
| Costing methodology | Whether sufficient details are given to make it possible to repeat the costing exercise, including the type of costing methodology used (e.g., marginal versus average costing methodology) |  |  |  |  |
| Currency, price date, and conversion | Report the dates of the estimated resource quantities and unit costs, plus the currency and year of conversion. |  |  |  |  |
| **RESOURCE IDENTIFICATION** | | | | | |
| Identification of resource elements | Explicit inclusion and exclusion criteria, adequate justification of exclusion and inclusion of any cost items |  |  |  |  |
| Methods/ tools used to identify resource items | Sources are explicitly stated, and methods are described (e.g., clinical care pathways, literature, etc.) |  |  |  |  |
| Sample size calculation | Described in sufficient details |  |  |  |  |
| Resource items are clearly identified and listed | Separate, detailed reporting of cost items by cost categories. Unit of measurement is clearly stated, and the quantities of resources are reported separately from their unit cost. |  |  |  |  |
| Classification of cost items | Direct costs, overheads, fixed, variable, etc. |  |  |  |  |
| Identification of joint costs | Dealing with costs attributed to co-morbid conditions |  |  |  |  |
| Subgroup analysis | Grouping patients by disease severity, prognosis, and/or co-morbidities |  |  |  |  |
| Assumption regarding resource use measurement | Describe any methods for analyzing or statistically transforming data and any extrapolation methods. |  |  |  |  |
| Dealing with uncertainties | Sensitivity analysis / statistical tests used. |  |  |  |  |
| **RESOURCE VALUATION** | | | | | |
| Valuing resource items | Clear description of the method and source of unit cost data |  |  |  |  |
| Direct measurement | Describe how costs were measured and valued. |  |  |  |  |
| Prices for marketable items | Competitive or Non-competitive market prices were used. |  |  |  |  |
| Fees/tariffs/charges | Justification is provided. |  |  |  |  |
| Sources of cost estimates | Explicitly stated (e.g., literature or other source) |  |  |  |  |
| Assumptions regarding attaching monetary value to resource use | Describe any methods for analyzing or statistically transforming data and any extrapolation methods. Further, any sensitivity analysis or assumptions applied in the study (e.g., practice pattern variation within a particular country and between countries) |  |  |  |  |
| **RESULTS** | | | | | |
| Study parameters | Report all analytic inputs (e.g., values, ranges, references) including uncertainty or distributional assumptions. |  |  |  |  |
| Summary of main results | Report the mean values for the main categories of costs and patient activities of interest and summaries them in the most appropriate overall measure. |  |  |  |  |
| Effect of uncertainty | Describe how uncertainty about analytic judgments, inputs, or projections affect findings. Report the effect of choice of discount rate and time horizon, if applicable. |  |  |  |  |
| Effect of health system-specific factors | Such as practice pattern variation, organization of health service delivery, financial arrangements (e.g., reimbursement/payment mechanism, shifting, existence of cost sharing), economic development / health care expenditures (e.g., total health care expenditures) |  |  |  |  |
| **DISCUSSION** | |  |  |  |  |
| Study findings, limitations, generalizability, and current knowledge | Report key findings, limitations, ethical or equity considerations not captured, and how these could impact patients, policy, or practice. |  |  |  |  |
| **OTHER RELEVANT INFORMATION** | | | | | |
| Source of funding | Describe how the study was funded and any role of the funder in the identification, design, conduct, and reporting of the analysis |  |  |  |  |
| Conflicts of interest | Report authors conflicts of interest according to journal or International Committee of Medical Journal Editors requirements. |  |  |  |  |
